# Biological Activities, Medicinal Uses, and Nutritional Effects of *Terminalia arjuna* (Roxb. ex DC.) Wight & Arn.: Phytochemical Insights and Therapeutic Benefits in Ulcerative Colitis Management—A Detailed Review

**DOI:** 10.1002/fsn3.72329

**Published:** 2026-09-22

**Authors:** Rameen Naeem, Muhammad Tauseef Sultan, Muhammad Usman Khalid, Muhammad Maaz, Ayman Furqan, Laiba Tanvir, Fabiano Travaglia, Matteo Bordiga, Mutjardah Zarrish

**Affiliations:** ^1^ Faculty of Food Science and Nutrition Bahauddin Zakariya University Multan Pakistan; ^2^ Department of Food Sciences, Faculty of Food, Nutrition and Home Sciences GIFT University Gujranwala Pakistan; ^3^ Department of Pharmaceutical Science Università Degli Studi del Piemonte Orientale “A. Avogadro” Novara Italy

**Keywords:** functional food, gastro‐protective, phytochemistry, *Terminalia arjuna*, ulcerative colitis

## Abstract

*Terminalia arjuna* (Roxb.) Wight & Arn, a medicinal tree, is extensively utilized in traditional Ayurvedic medicinal systems due to its pharmacological properties, predominantly anti‐inflammatory, antioxidant, immune‐modulatory, and cardio‐protective. However, its therapeutic potential in the management of ulcerative colitis (UC) is relatively less explored; therefore, this review was conducted to extensively evaluate the effect of *T. arjuna* and its phytoconstituents, like flavonoids, tannins, and triterpenoids, in the management of UC. Preclinical studies reveal that *T. arjuna* extracts and bioactive compounds, notably arjunolic acid, attenuate colonic inflammation by modulating inflammatory cytokines, antioxidant defense, restoring mucosal barrier integrity, and gut microbiota composition. Moreover, its cyto‐protective and free‐radical scavenging activities further enhance its adequacy for gastro‐protection. Furthermore, the pharmacological attributes suggest that it can play a promising role in food‐based interventions and gut‐health targeted products, significantly contributing to lowering inflammation. It is reported that *T. arjuna*‐enriched food products are effective towards plant‐based, natural therapies for chronic disorders, particularly UC. Despite various studies targeting its medicinal roles, the gastro‐protective and anti‐colitis potential of *T. arjuna* remains clinically underexplored, with preclinical evidence providing a promising but incomplete foundation. This review provides insights into existing data on therapeutic effects of *T. arjuna* in the management of UC and gastrointestinal health.

## Graphical abstract

This graphical abstract highlights the versatile therapeutic potential and food applications of *Terminalia arjuna*, centering on its broad health‐promoting bioactivities. Key mechanisms described include adaptogenic, cardioprotective, gastroprotective, wound‐healing, and anti‐inflammatory effects mediated through Nrf2 and NF‐κB signaling pathways alongside key pro‐inflammatory cytokines (TNF‐α, IL‐6, and IL‐1β). Ultimately, the image emphasizes its targeted role in mitigating ulcerative colitis by addressing immune dysfunction and cytokine imbalances alongside its utility in food science.

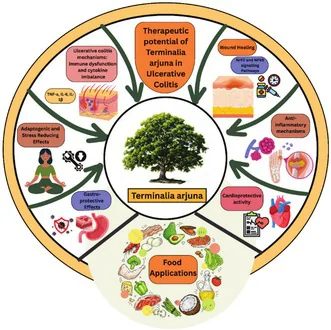

## 1 Introduction

Ulcerative colitis (UC) is an idiopathic and chronic condition, which mostly impacts the colonic mucosa (He et al. 2025). Clinical manifestations include chronic diarrhea, rectal bleeding, abdominal pain, and a comparatively increased risk of colorectal cancer (Gruber et al. 2024). The global incidence and prevalence of UC are increasing in developing nations undergoing rapid westernization of lifestyles, creating a significant public health burden (Kaplan 2025). The pathogenesis of UC involves a complex interplay of genetic factors, immune dysfunction, alterations in gut microbiota, and environmental or lifestyle triggers (Imamovna 2025). Regardless of the cause, inflammation in UC is always a result of an exaggerated immune response that involves excessive production of pro‐inflammatory cytokines such as tumor necrosis factor‐alpha (TNF‐α), reactive oxygen species (ROS), interleukin‐6 (IL‐6), and interleukin‐12 (IL‐12) (Alqudah et al. 2025).

The medical treatment of UC involves use of corticosteroids, aminosalicylates, biologics, and immunosuppressants that mainly aim to induce and maintain disease remission (Siddiqui et al. 2025). However, these therapies are mostly unable to achieve complete remission and include additional burdens of adverse side effects, high cost, and potential threat of developing resistance or immunogenicity (Chaemsupaphan et al. 2025). Moreover, despite these therapies, almost 15% of patients still struggle with relapses and eventually require complete or partial colectomies (Bækgaard et al. 2025). Due to perceived lack of response with standard therapies, there is increasing interest in herbal therapies and alternative medicine. Medicinal plants offer safer and multi‐targeted bioactivities for safer management of UC (Mutheeswaran et al. 2025). Among these, *Terminalia arjuna*, a native Indian deciduous tree, has a wide range of pharmacological properties, among which the prominent ones include antioxidant, anti‐inflammatory, and gastroprotective effects (Rajaram et al. 2024).

Historically, *T. arjuna* has been utilized in ayurvedic and traditional medicine systems primarily for cardiovascular ailments, but emerging evidence supports its potential role in gastrointestinal health and inflammatory disorders (Irfan et al. 2025). The bark of *T. arjuna* is rich in bioactive phytochemicals such as triterpenoids, flavonoids, tannins, and glycosides that exert immunomodulatory and cytoprotective effects (Tahir et al. 2025). Preclinical studies suggest that *T. arjuna* can attenuate colonic inflammation by downregulating pro‐inflammatory cytokines and oxidative stress markers thereby promoting mucosal healing and modulating gut microbiota (Cota et al. 2019). Nonetheless, evaluation of *T. arjuna*'s efficacy in UC remains scarce. This review has mainly focused on analyzing the existing literature on *T. arjuna*, with special emphasis on the plant's phytochemistry, pharmacological actions and therapeutic potential in UC.

## 2 Methods

The review was structured by conducting systematic searching from online databases, such as Google Scholar, PubMed, Science Direct, and Wiley Online Library. The papers were selected during the timeframe of 2000–2025. The following keywords were used: “Terminalia arjuna” OR “*T. arjuna*” OR “Arjuna bark” AND (“phytochemistry” OR “bioactive compounds” OR “arjunolic acid” OR “flavonoids” OR “tannins” OR “triterpenoids”) AND (“ulcerative colitis” OR “inflammatory bowel disease” OR “gastroprotection” OR “gut microbiota” OR “mucosal healing” OR “colonic inflammation”) AND (“antioxidant” OR “anti‐inflammatory” OR “antimicrobial” OR “hepatoprotective” OR “cardioprotective” OR “antidiabetic” OR “reno‐protective”) AND (“review” OR “meta‐analysis” OR “clinical trial” OR “original research”) AND NOT (“editorial” OR “conference abstract” OR “non‐peer reviewed”) and using Boolean operations (AND & OR).

Studies on *T. arjuna* phytochemistry, biological activities, pharmacological properties, and therapeutic applications in gastrointestinal and metabolic disorders were included along with original research and reviews published in English. Studies investigating other *Terminalia* species without direct comparison to *T. arjuna*, non‐English publications, conference abstracts without full‐text availability, and studies with insufficient methodological detail or weak evidence base were excluded. After screening the retrieved titles and abstracts, only studies meeting the inclusion criteria were selected for full‐text review and data extraction, which was organized thematically according to biological activity or disease condition investigated.

### 2.1 Taxonomical Classification

In addition to the standard botanical classification, medicinal plants are classified based on their habitat, part used, and therapeutic value, etc. Table 1 represents the taxonomical classification of *T. arjuna* (Hafiz et al. 2014).

**TABLE 1:** Taxonomical classification of *Terminalia arjuna*.

Kingdom	Plantae
Division	Magnoliphyta
Class	Magnoliopsida
Order	Myrtales
Family	Combretaceae
Genus	Terminalia
Species	*Terminalia arjuna* (Roxb.) Wight & Arn.

### 2.2 Botanical Features and Geographical Description

*Terminalia arjuna* is native to Asia, mostly located in semi‐arid, sub‐humid, and humid tropical belts, with a marked favorability for moist riparian habitats, stream banks, dry riverbeds, and ravines. Within India, common locations are from Punjab, Maharashtra, Uttar Pradesh, Himachal Pradesh, Tamil Nadu, South Bihar, West Bengal, Odisha, and Madhya Pradesh, as well as at the side of ponds and rivers in Delhi and the Deccan (Kumar et al. 2023). The species also extends into the plains of Pakistan and forests of Sri Lanka, Mauritius, and Burma/Myanmar (Rahman and Amin 2003).

**FIGURE 1:**
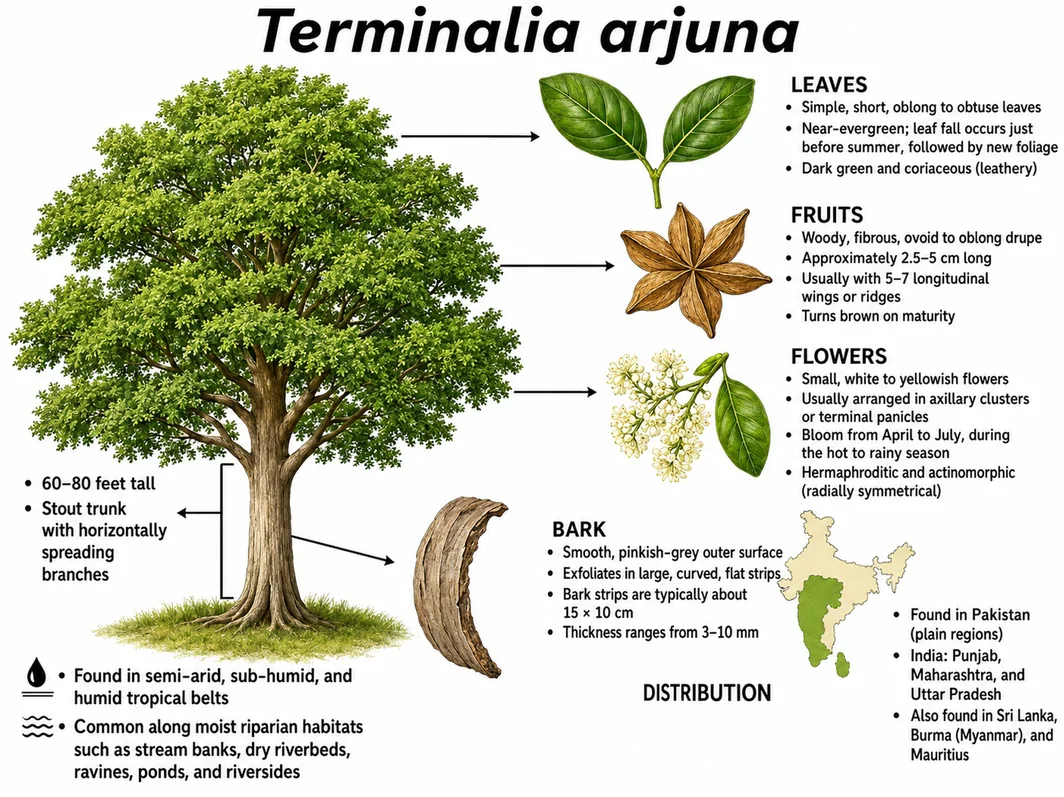
Botanical features of *Terminalia arjuna*.

Morphologically, *T. arjuna* is a tall, evergreen tree (60–80 ft) with a stout trunk and horizontally spreading branches; leaves are typically short with obtuse to oblong apices (Kapoor et al. 2014). The bark is diagnostically distinctive being smooth and pinkish‐gray externally, exfoliating in large, curved, flat strips (~15 × 10 cm), 3–10 mm thick (Rahman and Amin 2003). Ecologically, it grows in high‐moisture areas along with watercourses and plains, usually where yearly rainfall ranges from 750 to 1750 mm but persisting to ~3800 mm. The thermal tolerance of *T. arjuna* ranges from −1°C to 48°C, representing broad climatic adaptability (Dwivedi 2007). Local distribution is strongly constrained by near‐root moisture availability (Luna et al. 2011). Edaphically, *T. arjuna* thrives in deep, fertile, well‐drained alluvial loams, yet it can be grown on acidic red soils and even highly alkaline areas (pH ≤ 9.7) when moisture is adequate (Roodagi 2015). Phenologically, it is near evergreen, with leaf abscission preceding a flush of new foliage in the early hot season (Rahman and Amin 2003). The botanical features of *T. arjuna* are shown below in Figure 1.

### 2.3 Nutritional Composition and Phytochemistry

The therapeutic and economic value of *T. arjuna* tree is ascribed to different bioactive compounds that exhibit a beneficial role in both humans and animals. Numerous phytochemicals like tannins, flavonoids, glycosides, and terpenoids from *T. arjuna* bark have been reported to have medicinal properties (Abo‐Elghiet et al. 2022; Stępnik et al. 2023). The proximate composition of *T. arjuna* bark varies across different studies; moisture ranges from approximately 6.1%–10.9% (Meena et al. 2024; Goel and Tarvinderjeet 2013); total ash content spans 3.467%–17.49% (Wadhai et al. 2025; Chaudhari and Mahajan 2015), ether extract (fat) content varies between 0.53% and 1.9%, and 4.4%; crude protein ranges from 2.8% to 4.1%, and crude fiber falls between 7.6% and 9.79% (Meena et al. 2024; Goel and Tarvinderjeet 2013). Meanwhile, carbohydrate content is reported around 48.5%–66.1%, constituting the major proportion of the bark's composition. These variations reflect differences in analytical methods and extraction conditions yet collectively emphasize the bark's rich nutritional profile. Table 2 represents the nutritional composition of *T. arjuna*.

**TABLE 2:** Nutritional composition of *Terminalia arjuna*.

Plant part	Analyte	Value (unit)	Basis/Method	References
Bark	Moisture	7.1%	Sun‐dried bark, proximate analysis for “nutra tea” ingredient	Gorde and Ganjale (2020)
Bark	Crude protein	11.94% (summer, apical bark); 4.34% (winter, apical bark)	Seasonal and tissue variation across apical/middle/mature inner bark; biochemical assays	Patil and Gaikwad (2011)
Bark	Crude fiber	12.3%–18.9%	Range across bark regions/seasons	Patil and Gaikwad (2011)
Bark	Total ash	19.0%–31.3%	Higher in mature inner bark; seasonal effect	Patil and Gaikwad (2011)
ark	Total ash	14.5%	Independent proximate panel (tea formulation study)	Gorde and Ganjale (2020)
Bark	Total dietary fiber	21.6%	Same proximate panel	Gorde and Ganjale (2020)
Bark (powder)	Calcium (Ca)	34.1 ± 10.6 mg/g	Instrumental Neutron Activation Analysis (INAA) across 5 commercial/field powders	Garg et al. (2017)
Bark (powder)	Magnesium (Mg)	5.41 ± 1.93 mg/g	INAA	Garg et al. (2017)
Bark (powder)	Potassium (K)	5.87 ± 2.18 mg/g	INAA	Garg et al. (2017)
Bark (powder)	Iron (Fe)	2.99 ± 1.77 mg/g	INAA	Garg et al. (2017)
Bark (literature)	Ash	~34%	Historic/pharmacognosy accounts; largely calcium carbonate	Amalraj and Gopi (2017)
Bark (quality spec)	Loss on drying	3.28%	Pharmacopeial/WHO style quality assessment of stem bark	Kumar et al. (2016)
Fruit—pericarp (PC + SC)	Total polyphenols	2543 mg rutin hydrate/100 g	Fruits harvested in Raipur (India)	Quesada‐Granados et al. (2023)
Fruit—pericarp (PC + SC)	Iron (Fe)	35.84 mg/100 g	Same dataset; pericarp high in Fe	Quesada‐Granados et al. (2023)
Fruit—pericarp (PC + SC)	Copper (Cu)	181.64 mg/100 g	Highest Cu among samples analyzed	Quesada‐Granados et al. (2023)
Leaf (powder)	Crude protein	13.66%	Included sparingly from a comparative study (for leaves only)	Meena et al. (2020)
Leaf (powder)	Ash	11.48%	Same	Meena et al. (2020)

Abbreviations: Ca, calcium; Cu, copper; Fe, iron; INAA, instrumental neutron activation analysis; K, potassium; Mg, magnesium; PC + SC, pericarp + seed coat; WHO, World Health Organization.

Moreover, the concentration of aqueous extract of *T. arjuna* is 23% calcium salts and 16% tannins compared to the alcoholic extract which shows a lower concentration of coloring matter and tannins (Tahir et al. 2025). Flavonoids, like quercetin, have been shown to have a high antioxidant effect among them, and triterpenoid saponins, including arjunic acid and arjunolic acid, have been mentioned due to their cardioprotective effect (Tahsin et al. 2021). In fact, phytochemical screening revealed *T. arjuna* to possess numerous bioactive constituents such as phytosterols, lactones and phenolic compounds which have been found to contribute towards its antioxidant and anti‐inflammatory activity (Mishra et al. 2025). Moreover, these chemicals assist in reducing oxidative stress that is linked to several chronic illnesses because of scavenging reactive oxygen species (ROS) (Chatha et al. 2014). In particular, arjunolic acid was reported to possess considerable cardioprotective and antioxidant properties, which confirms that the substance can be used in ayurvedic medicine to combat cardiovascular diseases (Gouthamchandra et al. 2024; Ghosh and Sil 2013).

Among flavonoids, *T. arjuna* constitutes arjunone, luteolin, baicalein, kaempferol, and quercetin (Sahoo 2022). These flavonoids have elicited tremendous attention due to their antioxidant effect, anti‐inflammatory, and anti‐atherosclerotic effects. Specifically, quercetin is reported to possess a high degree of antioxidant potential, which is positively related to the decrease of oxidative stress, a significant contributor to the occurrence of cardiovascular diseases (Papakyriakopoulou et al. 2022; Subramaniam et al. 2011; Sivalokanathan et al. 2006). Moreover, tannins are another group of phytochemicals present in *T. arjuna*. Much attention has been given to the tannins that can be hydrolyzed, such as casuarinin, punicalagin, and arjunin (Tahir et al. 2025). Punicalagin, a significant ellagitannin widely studied in *Punica granatum* and also identified in *T. arjuna*, has been associated with potent antioxidant activity and is able to neutralize ROS to offer protection against cellular damage (Swain et al. 2023). Besides, it has been demonstrated that *T. arjuna* contains high levels of polyphenolic compounds, including gallic acid and ellagic acid, which have demonstrated high antioxidative and cardioprotective properties, justifying traditional medicinal beliefs (Ramesh and Palaniappan 2023). The phytochemistry of *T. arjuna* is elaborated below in Table 3.

**TABLE 3:** Phytochemistry of *Terminalia arjuna*.

Plant part/matrix	Phytochemical class	Representative compounds	References
Bark (stem)	Pentacyclic triterpenoids (oleanane/ursane, glycosides)	Arjunolic acid; Arjunic acid; Arjungenin; Arjunetin; Arjunglucosides I–V; Arjunasides A–E; β‐Sitosterol; Daucosterol	Wang et al. (2010)
Bark (stem)	Phenolics and ellagitannins; proanthocyanidins; flavan‐3‐ols	Gallic acid; Ellagic acid; 3‐O‐methyl‐ellagic acid 4‐O‐β‐D‐xylopyranoside; 3‐O‐methyl‐ellagic acid 3‐O‐rhamnoside; (+)‐Catechin; (+)‐Gallocatechin; (−)‐Epigallocatechin; Proanthocyanidins	Saha et al. (2012)
Bark (stem)	Triterpenoid acids (quantification/analytical)	Arjunolic acid; Arjungenin (HPTLC quantification in stem bark)	Kalola and Rajani (2006)
Bark (stem)	Triterpenoid acids (quantification/analytical)	Arjunic acid; Arjunolic acid (HPLC‐PDA quantification; MAE extraction)	Verma, Pal, et al. (2025)
Leaves	Flavonoids and phenolic acids (multi‐part profiling)	Quercetin; Luteolin; Apigenin; Myricetin; Gallic acid; Ellagic acid (reported across plant parts)	Singh et al. (2016); Ramesh and Palaniappan (2023)
Leaves	Tannins, saponins, steroids (qualitative screening)	Presence of tannins/phenolics, steroids, alkaloids, flavonoids, saponins (solvent‐dependent)	Meena et al. (2021)
Fruits	Tannins/phenolics, alkaloids, steroids, saponins (qualitative)	Qualitative detection of major classes; relative enrichment varies by solvent fraction	Meena et al. (2021)
Flowers	Volatile/phenolic profile by GC–MS; antioxidant and anticholinesterase activity	Diverse phenolics, esters and fatty‐acid derivatives reported in alcoholic extracts (region‐specific collection)	Elwekeel et al. (2024)
Heartwood (core wood)	Pentacyclic triterpenoid	Arjunolic acid (isolation/characterization; bioactivity assays)	Ramesh et al. (2012); Toppo et al. (2018)

Abbreviations: GC–MS, gas chromatography–mass spectrometry; HPLC–PDA, high‐performance liquid chromatography with photodiode array detection; HPTLC, high‐performance thin‐layer chromatography; MAE, microwave‐assisted extraction.

### 2.4 Traditional and Medicinal Attributes of *T. arjuna*

In ayurvedic systems, *T. arjuna* powders and decoctions are widely utilized for the treatment and cure of symptoms associated with ischemic heart disease (IHD) and congestive heart failure (CHF) (Dutta and Das 2023). In addition, *T. arjuna* is used to manage asthma, anemia, and bronchitis, which is justified by the fact that it has an anti‐inflammatory, antioxidant, and rejuvenating effect (Jude et al. 2022). Not only this, *T. arjuna* can be utilized in wound treatment through the topical application of the powdered bark preparations because of its antimicrobial, anti‐inflammatory, and astringent effects (Amalraj and Gopi 2017). Furthermore, it is also used in the treatment of gastrointestinal conditions such as diarrhea, dysentery, and peptic ulcers because of its strong gastroprotective and antibacterial potential (Dwivedi and Chopra 2014).

*Terminalia arjuna* has also been applied in the treatment of diabetes, obesity, and high cholesterol levels due to its traditional medicinal application, known as lipid‐lowering and hypoglycemic effects (Chaudhary and Sharma 2020; Subramaniam et al. 2011). Also, *T. arjuna* has been found to be effective in skin diseases, UTI, hepatic functions, and polymorphic pharmacological activity in conventional medicine (Amalraj and Gopi 2017; Mittal et al. 2016). Table 4 shows the medicinal properties and health benefits of *T. arjuna*.

**TABLE 4:** Medicinal attributes and health benefits of *Terminalia arjuna*.

Category	Indication/model	Population and design	Dose/form (duration)	Key findings	References
Cardioprotective	Chronic stable angina	58 adults; randomized, double‐blind, crossover vs. placebo and isosorbide dinitrate	Bark extract 500 mg q8h (4 weeks/phase)	↓ angina frequency and nitrate use; improved treadmill parameters vs. placebo	Bharani et al. (2002)
	Chronic heart failure	56 CHF pts.; randomized, double‐blind, placebo‐controlled	Standardized bark extract (12 weeks)	No LVEF change; some gains in functional capacity, antioxidant status & QoL domains.	Maulik et al. (2016)
	Platelet aggregation/atherothrombosis (adjunct)	CAD pts.; clinical summary + small trials	Bark extract (varied)	Signals for ↓ platelet aggregation & BP with favorable lipids; small‐study evidence base.	Priya et al. (2019)
	Mechanistic anti‐atherogenic	In vitro macrophage foam‐cell model	Bark extract (standardized)	Reduced foam‐cell formation; promoted apoptosis via UPR‐CHOP pathway.	Bhansali et al. (2019)
	Thyroid‐linked (cardio‐protection mechanistic)	Rats	Bark extract (ethanolic)	Cardio‐protection possibly mediated by altered thyroid hormones.	Parmar et al. (2006)
Gastroprotective	NSAID‐induced ulcer	Rats (diclofenac model)	Methanolic bark extract	↓ lesion index; increased mucosal protection.	Devi et al. (2007)
	*H. pylori* LPS‐induced gastric damage	Rats	Methanolic bark extract	Significant anti‐ulcer effect vs. controls.	Devi et al. (2008)
	Ethanol/HCl & pylorus‐ligated ulcers	Rats	Methanolic bark extract	↑ adherent mucus; ↑ protein‐bound carbohydrates; anti‐ulcer and hypokinetic activity.	Nunes et al. (2014)
Reno‐protective	Oxidative stress in isolated kidney	Isolated perfused rat kidney	Bark extract (ex vivo)	Improved renal antioxidant milieu.	Raj et al. (2012)
	Cisplatin nephrotoxicity	Rats	Arjunolic acid (from *T. arjuna*)	Attenuated cisplatin‐induced renal injury and oxidative stress.	Elsherbiny et al. (2016)
	Dehydration‐induced oxidative stress (renal)	Rats	Aqueous bark extract	Protected against dehydration‐related oxidative stress; reno‐protective signals.	Das et al. (2010)
Anti‐diabetic/metabolic	Type 2 diabetes—clinical	Adults with T2DM; randomized comparative vs. sitagliptin	Standardized bark formulation (~1 g/day; 12 weeks)	↓ fasting plasma glucose & HbA1c; favorable safety pilot RCT	Prakash et al. (2024)
	DPP‐IV inhibitory & HbA1c	Adults with diabetes (exploratory)	Standardized bark extract	DPP‐IV inhibition with reductions in HbA1c reported; needs robust replication	Mohanty, Borde, Kumar, and Maheshwari (2019) and Mohanty, Borde, and Maheshwari (2019)
	HFD/STZ diabetes model	Rats	Bark extract	↓ hyperglycemia, dyslipidemia & oxidative stress; improved hepatic/pancreatic antioxidants	Parveen et al. (2011)
Hepatoprotective	NAFLD models	Cells & rodents	Arjunolic acid (isolated from *T. arjuna*)	Ameliorated steatosis and liver injury in NAFLD models	Toppo et al. (2018)
	Isoniazid liver injury	Rats	Bark powder	Lowered AST/ALT vs. toxin control; hepatoprotection suggested	Iqram et al. (2025a, 2025b)
Other uses	Antimicrobial activity	In vitro; multiple pathogens	Bark & leaf extracts	Broad‐spectrum antibacterial activity demonstrated	Aneja et al. (2015)
	Anti‐inflammatory	Rats carrageenan (paw edema)	Aqueous bark extract	Dose‐dependent anti‐inflammatory activity	Dube et al. (2017); Rana et al. (2016)
	Anti‐arthritic	Rats (collagen‐induced arthritis)	Bark extract	Reduced oxidative stress & disease activity in CIA model	Tyagi and Khan (2018)
	Chronic venous insufficiency (CVI)	Adults; prospective observational	Oral *T. arjuna* preparation	Improved CVI symptoms and ulcer healing; safety acceptable.	Shankar (2024)

Abbreviations: ALT, alanine aminotransferase; AST, aspartate aminotransferase; BP, blood pressure; CAD, coronary artery disease; CHF, congestive heart failure; CIA, collagen‐induced arthritis; CVI, chronic venous insufficiency; DPP‐IV, dipeptidyl peptidase‐4; HbA1c, glycated hemoglobin; HFD, high‐fat diet; LPS, lipopolysaccharide; LVEF, left ventricular ejection fraction; NAFLD, non‐alcoholic fatty liver disease; NSAID, non‐steroidal anti‐inflammatory drug; QoL, quality of life; RCT, randomized controlled trial; STZ, streptozotocin; *T. arjuna*, *Terminalia arjuna*; T2DM, Type 2 diabetes mellitus; UPR‐CHOP, unfolded protein response–C/EBP homologous protein.

### 2.5 Ulcerative Colitis Mechanisms

#### 2.5.1 Immune Dysregulation and Cytokine Imbalance

The immune system plays a key role in the development and maintenance of UC. In physiological conditions, intestinal epithelial cells bear commensal microorganisms and cause relevant and needed responses to pathogens. This balance is disrupted in UC, leading to abnormal immune reactions to luminal antigens. Naïve T‐cells are first activated into effector cells by antigen‐presenting cells, and Th‐1 and Th‐17 are primarily involved in the prognosis of UC (Tindemans et al. 2020). These cells generate great amounts of pro‐inflammatory cytokines such as tumor necrosis factor‐alpha (TNF‐α), interleukin‐1 beta (IL‐1β), interleukin‐6 (IL‐6), interleukin‐17 (IL‐17), and chemokines such as MCP‐1. This cytokine cascade maintains the inflammatory process, which attracts more neutrophils and leads to further damage to the epithelium (Neurath 2014).

Several studies have shown that *T. arjuna* was able to regulate cytokines. Moreover, *T. arjuna* hydroalcoholic extract (TAHA) has a strong effect of alleviating TNBS‐induced colitis in rats as it could reduce the disease activity index, macroscopic destruction, and histological indices. The administration of TAHA has been shown to markedly repress the mucosal expression of key pro‐inflammatory cytokines like TNF‐α, IL‐6, IL‐1β, and MCP‐1 (Cota et al. 2019). The overlap between these cytokine targets and those inhibited by biologics including infliximab or adalimumab highlights the potential of *T. arjuna* as a natural agent that may complement pharmacological options. Evidence from another study indicates that Arjunarishta, a fermented *T. arjuna* preparation, mitigated colonic inflammation and restored both histological structures and mucosal barrier integrity in experimental colitis (Cota et al. 2020).

**FIGURE 2:**
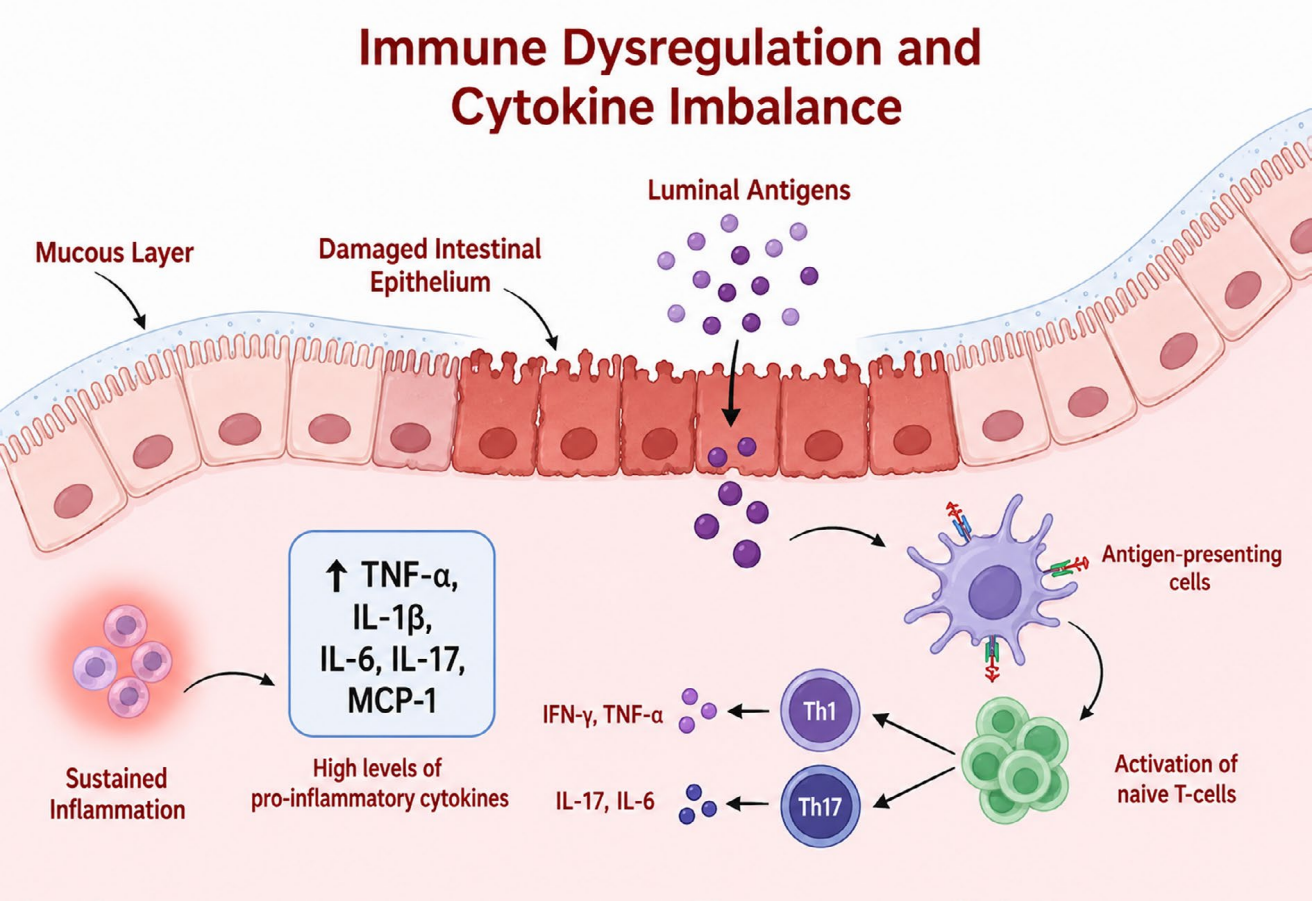
Immune dysregulation and cytokine imbalance in UC.

*Terminalia arjuna* inhibits a major pathway in UC by suppressing significant inflammatory mediators. The mechanism in which it inhibits the cytokine activity is similar to the mode of action of biologics, yet it may have fewer side effects. The immune dysregulation and cytokine imbalance that occurs in UC is shown in Figure 2.

#### 2.5.2 Antioxidant Defense and Oxidative Stress

Antioxidant activity defines the balance between free radicals and antioxidant mechanisms, thereby attenuating oxidative stress‐induced disorders (Maaz et al. 2025). Oxidative stress plays an important role in facilitating mucosal damage in UC. When the disease progresses, activated neutrophils and macrophages cause significant amounts of reactive oxygen species (ROS) and reactive nitrogen species (RNS) (Muro et al. 2024). These mediators of the inflammatory cascade create lipid peroxidation and structural damage on proteins and nucleic acids, which drives and prolongs the inflammatory response in chronic UC (Basak et al. 2020).

UC is also described as a disrupted antioxidant defenses especially lessened SOD, catalase, and GSH, which increase the risk to oxidative harm (Rana et al. 2014). *Terminalia arjuna* has elevated levels of polyphenols, flavonoids, tannins, and triterpenoids including arjunolic acid, which has a strong antioxidant potential (Sahoo 2022). Results of the TNBS‐induced colitis models indicate that TAHA significantly reduces the presence of oxidative biomarkers (MDA, MPO, NO) and replenishes major antioxidant enzymes (SOD, catalase, GSH). The clinical significance of such redox restoration is proven, with interventions based on antioxidants proving beneficial in the mitigation of colonic damage in UC (Blagov et al. 2023).

**FIGURE 3:**
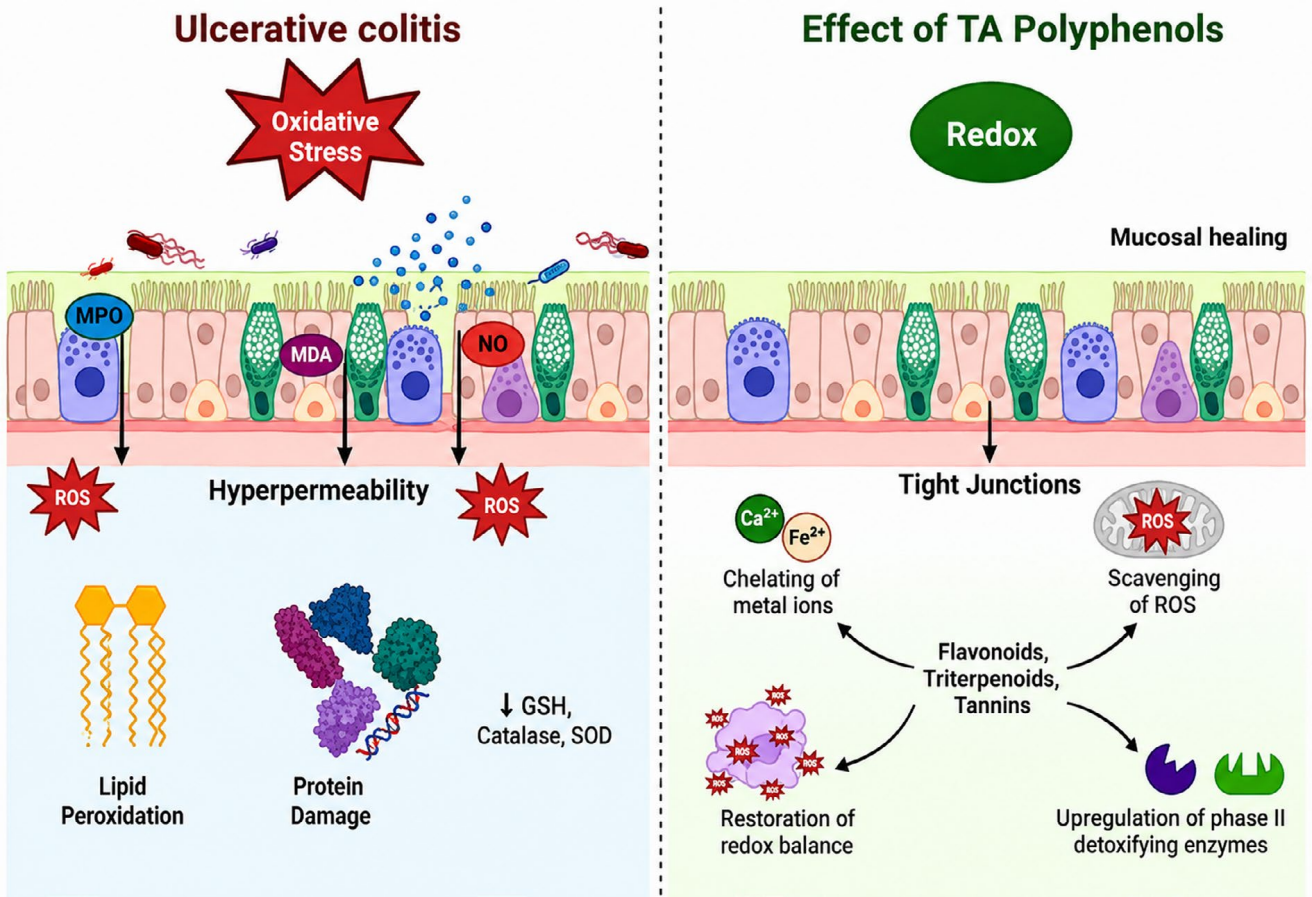
Oxidative stress in UC versus antioxidant defense by *Terminalia arjuna* phytochemicals.

Additionally, *T. arjuna's* flavonoids can sequester pro‐oxidant metal ions, neutralize reactive radicals, and activate the Nrf2 pathway to stimulate Phase II detoxification enzymes (Bhatia et al. 2014). *Terminalia arjuna* may assist in acute symptom relief and long‐term mucosal integrity by breaking the self‐perpetuating cycle of oxidative stress and epithelium degradation through these combined pathways. Figure 3 shows the oxidative damage occurring in UC and the effect of *T. arjuna* polyphenols in improving antioxidant defense.

#### 2.5.3 Barrier Dysfunction and Mucosal Healing

The disruption of the intestine epithelial barrier is one of the primary pathological features of UC. The integrity of the barrier requires coordinated tight junctions and adequate mucus production. The long‐lasting inflammation in UC raises permeability and antigen transfer because it leads to tight junction disintegration, fostering epithelial cell death and reducing the thickness of the mucus layer (van der Post et al. 2019). This compromised wall begins a self‐perpetuating inflammatory cascade and sustained immunological response.

It is increasingly evident that *T. arjuna* can enhance the action of the epithelial barrier. Colitis models demonstrate the enhancement of mucosal architecture, the decrease in ulceration, and the restoration of crypt structure by *T. arjuna* therapy (Cota et al. 2019). Since it has been shown to raise plasma zinc levels and zinc is required for tight junction integrity and epithelium regeneration (Rybakovsky et al. 2017), zinc‐mediated repair is one of the potential mechanisms. Moreover, *T. arjuna* can also be used to facilitate barrier restoration as well as its antioxidant and anti‐inflammatory actions, as mucosal repair, long‐term remission, and reduced risk of colorectal cancer have a high correlation, which is an important treatment aim in UC (Pineton de Chambrun et al. 2010).

#### 2.5.4 Gut Microbiota Modulation

The gut microbiota plays an important part in the maintenance of immune homeostasis as it is required to support epithelial barrier activity and nutrient absorption. Reduced microbial richness and the loss of beneficial microflora like *Faecalibacterium prausnitzii* and excess of potentially harmful bacteria is a common finding in UC (Ni et al. 2017). These changes impair epithelial energy metabolism, interfere with short‐chain fatty acid (SCFA) synthesis, and cause inflammatory signaling via pattern‐recognition receptors, such as toll‐like receptors (TLRs), which prolong mucosal inflammation (Liu et al. 2023).

The overall composition of intestinal microbes is regulated by *T. arjuna*. For instance, arjunarishta therapy decreased pathogenic microorganisms and raised the relative abundance of anti‐inflammatory taxa in colitis models (Cota et al. 2020). Its antioxidant, anti‐inflammatory, and barrier‐protective properties probably work in concert with this microbiota‐modulating characteristic. *Terminalia arjuna*'*s* phytochemicals and polyphenols may function as natural prebiotics, promoting beneficial microbial populations while preventing pathogenic proliferation, given the therapeutic promise of microbiota‐targeted therapies in UC.

#### 2.5.5 Nrf2 and NF‐κB Signaling Pathways

Inflammation is the body's first defense mechanism in response to foreign particles; however, the increased deleterious pathogens further enhance inflammation at dangerous levels (Noman et al. 2025). Dysregulation of transcription factors that control oxidative stress and inflammation shapes UC etiology at the molecular level. While nuclear factor erythroid 2–related factor 2 (Nrf2) promotes the production of cytoprotective and antioxidant genes, nuclear factor kappa‐B (NF‐κB) is the main regulator of pro‐inflammatory gene expression. Uncontrolled inflammation and oxidative damage are caused by UC's prolonged activation of NF‐κB and suppression of Nrf2 activity (Li et al. 2019).

**FIGURE 4:**
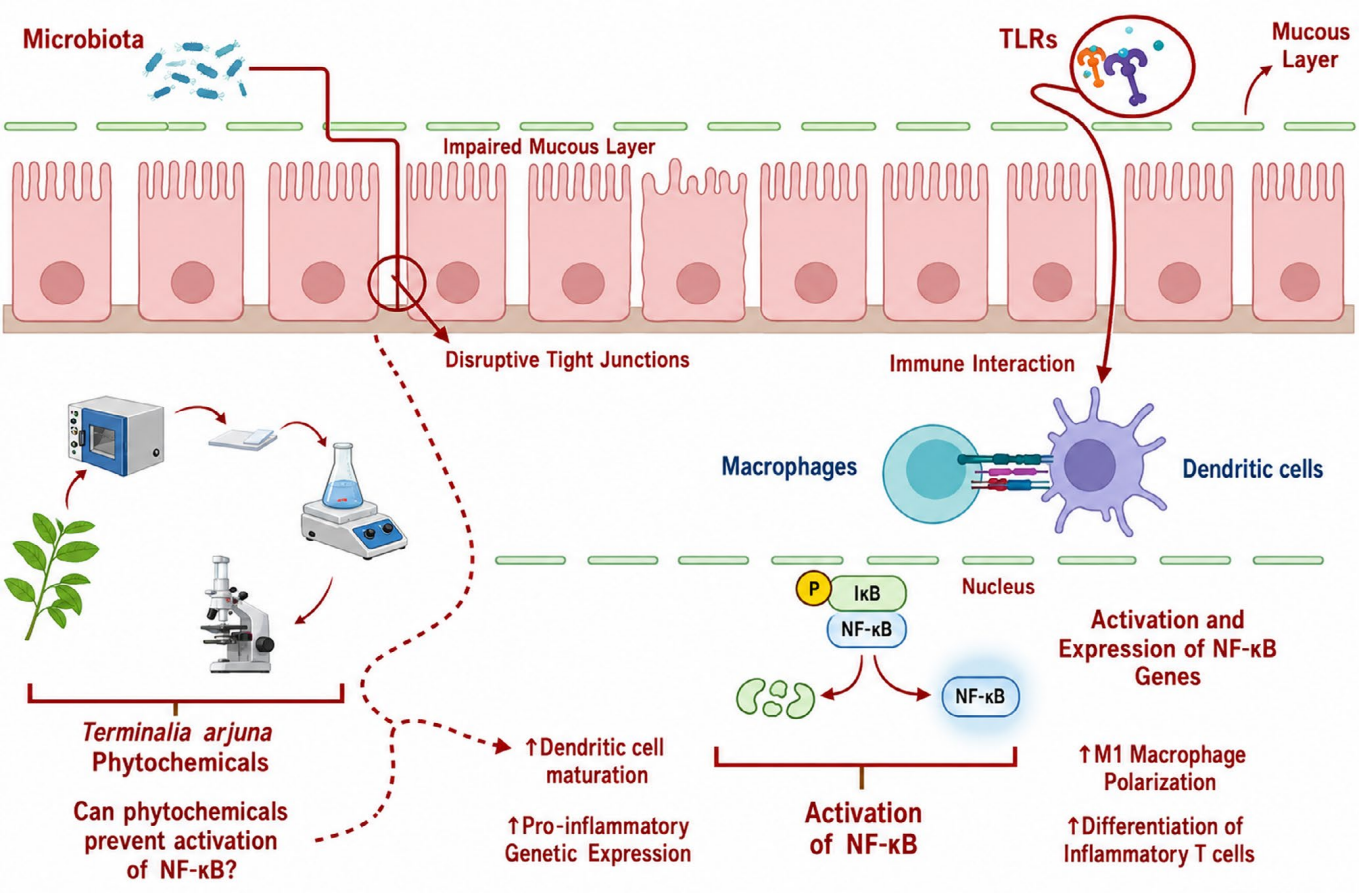
Nrf2 and NF‐κB signaling pathways.

Arjunolic acid, a triterpenoid derived from *T. arjuna*, has been shown to stimulate Nrf2 signaling while decreasing NF‐κB pathways in cardiovascular and hepatic models (Bhatia et al. 2014). The reported antioxidant and anti‐inflammatory benefits of *T. arjuna* are compatible with regulation of the Nrf2–NF‐κB axis, despite the lack of direct proof in UC models. *Terminalia arjuna* may provide a second strategy for lowering oxidative stress and inflammation by indirectly suppressing NF‐κB activation through increased Nrf2 activity (Ramesh and Palaniappan 2023). Figure 4 represents the Nrf2 and NF‐κB signaling pathways.

### 2.6 Role in Ulcerative Colitis

*Terminalia arjuna* has shown promising potential for managing UC that is a disease characterized by persistent inflammation and ulceration of the colon. Because UC is driven by multiple interconnected factors like oxidative stress, imbalance of the gut microbiome, and high pro‐inflammatory cytokines, treating it has remained a challenge (Ungaro et al. 2017). In this context, *T. arjuna* becomes an important therapeutic option due to its prominent antioxidant properties. Furthermore, studies have shown that *T. arjuna* bark extracts (100–200 mg/kg) can reduce colon shortening, mucosal ulceration, and histological damage in DSS‐induced colitis models. These effects are also linked with low TNF‐α, IL‐1β, and IL‐6 levels, as well as high colonic antioxidant enzyme activity (Khan et al. 2022).

Another study conducted by Sharma (2022) also assessed the use of arjunolic acid extracted out of *T. arjuna* in a rat model. They orally administered arjunolic acid in 50 mg/kg/day doses. Their findings involved a decrease in oxidative stress effectors like malondialdehyde and inflammatory cytokines that were abated like TNF‐α, IL‐17, and IL‐1β, which are inflammatory. Moreover, colonic glutathione levels were also restored by this dose. In addition, histopathological analysis indicated better mucosal healing, less crypt damage, and decreased inflammatory cell infiltration. Arjunolic acid is thus an effective natural therapeutic candidate in the treatment of UC.

Human studies have also investigated the clinical advantages of *T. arjuna* bark supplementation on the functional gastrointestinal diseases including persistent gastritis and irritable bowel syndrome (IBS). In their clinical trial, Kapoor et al. (2014) involved 80 patients with chronic gastritis and observed that the patients who were treated with standardized aqueous *T. arjuna* bark extract (500 mg in capsules, twice daily during 8 weeks) had significant improvement of their symptoms such as bloating, abdominal discomfort, and epigastric pain compared to placebo. These improvements were verified with endoscopic analyses that revealed better mucosal integrity and less mucosal erythema which favors the use of *T. arjuna* as a possible adjunct treatment of chronic gastritis.

Also, *T. arjuna* has been useful in the treatment of mucositis (a condition caused by chemotherapy). Mukherjee (2020) studied the preventive action of the *T. arjuna* ethanolic bark extract against methotrexate‐induced intestinal mucositis in rats. Pretreatment with 400 mg/kg of bark extract was successful in reducing mucosal injury, intestinal inflammation, and disruption of the epithelial barrier. These were biochemically mediated by increased antioxidant status in the intestinal mucosa and inhibition of NF‐κB activation that led to decreased production of inflammatory cytokines including TNF‐α and IL‐6. Moreover, *T. arjuna* bark extract acts by a set of biological mechanisms, such as by cytokine regulation, anti‐inflammatory pro‐mediator production, such as prostaglandins, and prevention of oxidative stress by free radical scavenging. Such measures reduce inflammation but do not result in any additional negative effects commonly associated with corticosteroids and NSAIDs (Huddar et al. 2024).

Preclinical evidence underscores the relevance of *T. arjuna* in colitis management, particularly by focusing on its whole extract and multi‐component formulations. In TNBS‐induced rat colitis, a hydro‐alcoholic *T. arjuna* bark extract reduced disease activity and histopathological injury, lowered colonic TNF‐α, IL‐1β, and IL‐6 levels, decreased oxidative stress markers, and partially normalized gut microbiota composition, aligning with key UC therapeutic goals. Similarly, a polyherbal formulation dominated by *T. arjuna* (Arjunarishta) produced comparable benefits in the same model, although the effects cannot be attributed solely to *T. arjuna* (Cota et al. 2020). Taken together, these findings justify systematic evaluation of standardized *T. arjuna* extract in UC, while underscoring that rigorously designed clinical trials in UC patients are still lacking.

Moving beyond whole‐plant extracts, researchers have also begun to unpack the specific biochemical mechanisms through which *T. arjuna* exerts its anti‐colitic effects. A detailed analysis of *T. arjuna* bark extract in TNBS colitis models revealed a remarkably broad mechanistic fingerprint in which it simultaneously down‐regulated key pro‐inflammatory cytokines (TNF‐α, IL‐1β, IL‐6 and MCP‐1), restored antioxidant defenses (↑SOD, CAT, GSH; ↓MPO, MDA, NO), shifted the fecal microbiota towards a more favorable composition, and elevated plasma zinc levels, the latter being particularly relevant to mucosal repair in UC (Cota et al. 2022). Drilling down further to the constituent level, the isolated triterpenoid arjunolic acid has shown notable protective effects in Crohn's disease‐like colitis, preserving epithelial barrier integrity by curbing apoptosis, dampening TLR4 signaling, and reshaping gut microbiota pathways that are equally implicated in UC‐related barrier failure (Zhang et al. 2024). Together, these findings begin to map out the molecular landscape through which *T. arjuna* operates, offering a more mechanistic rationale for its therapeutic potential in inflammatory bowel conditions.

**FIGURE 5:**
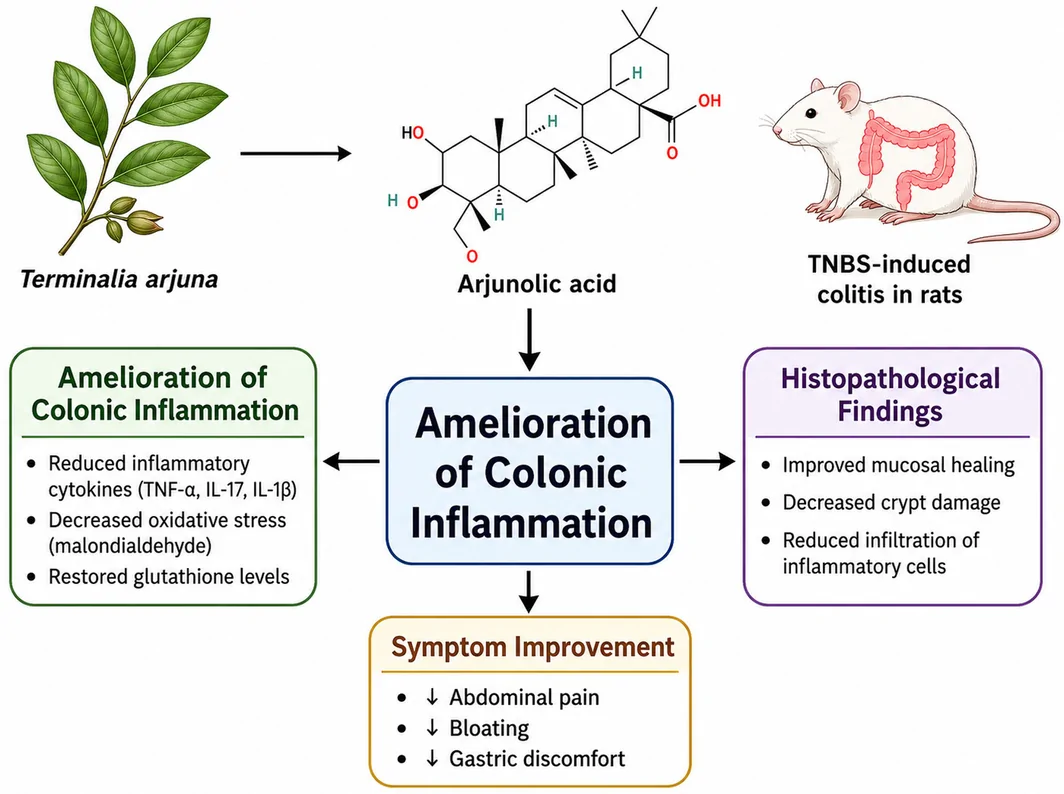
Role of *Terminalia arjuna* in treatment and management of UC.

Preclinical colitis studies and broader anti‐inflammatory data suggest that *T. arjuna* through triterpenoid‐rich extracts and arjunolic acid targets oxidative stress, innate immune activation (TLR4/NF‐κB), and epithelial‐barrier integrity; however, clinical trials in patients with UC are presently lacking. Figure 5 shows the role of *T. arjuna* in the treatment and management of UC.

### 2.7 Antioxidant Properties

*Terminalia arjuna* exhibits remarkable antioxidant effects that are mainly attributed to its rich phytochemical profile that includes powerful antioxidants like flavonoids, triterpenoids, and tannins. In animal studies, hydroalcoholic bark extract at 250–500 mg/kg reduced lipid peroxidation and normalized liver and kidney enzymes disrupted by oxidative stress. Enhancement of antioxidant defenses, such as catalase and glutathione peroxidase, is responsible for this impact (Raj et al. 2012). Important phytochemicals that reduce oxidative stress and inflammation linked to cardiovascular disease include arjunolic acid and arjungenin, which further decrease nitric oxide generation in inflammatory cells (Grewal et al. 2023).

In recent studies, *T. arjuna* alcoholic extracts were found to possess good antioxidant activity in different tests, such as lipid peroxidation, superoxide scavenging, and DPPH radical‐scavenging assays (Singh et al. 2025). The ethanol bark extract contained the highest amounts of phenolic and flavonoid compounds and had the ability to protect DNA against the damage of hydrogen peroxide, as well. This DNA‐protective activity indicates its possible role in preventing oxidative genetic damage, which is one of the primary contributors to aging and disease development (Meena et al. 2021).

Similarly, *T. arjuna* stem bark extract (ALTA) is a powerful antioxidant extract containing a wide variety of polyphenols, flavonoids, tannins and triterpenoids including arjunolic acid. It has good free radical scavenging activity, having very low EC50 in DPPH and other assays, and at the same time boosting endogenous antioxidant defenses. ALTA also protects important enzymatic antioxidants, including superoxide dismutase, catalase, and glutathione peroxidase, as well as non‐enzymatic, including glutathione, and vitamin C, and decreases oxidative stress indicators, such as lipid peroxidation and myeloperoxidase. Also, it shields the DNA against oxidative effects and prevents inflammation by inhibiting the formation of nitric oxide, which is especially important to the health of the heart. These protective effects are further corroborated by preclinical studies, which reveal that oxidative injury of the heart and kidneys is mitigated with the compound (Vijayalakshmi et al. 2023; Hasan et al. 2023).

**FIGURE 6:**
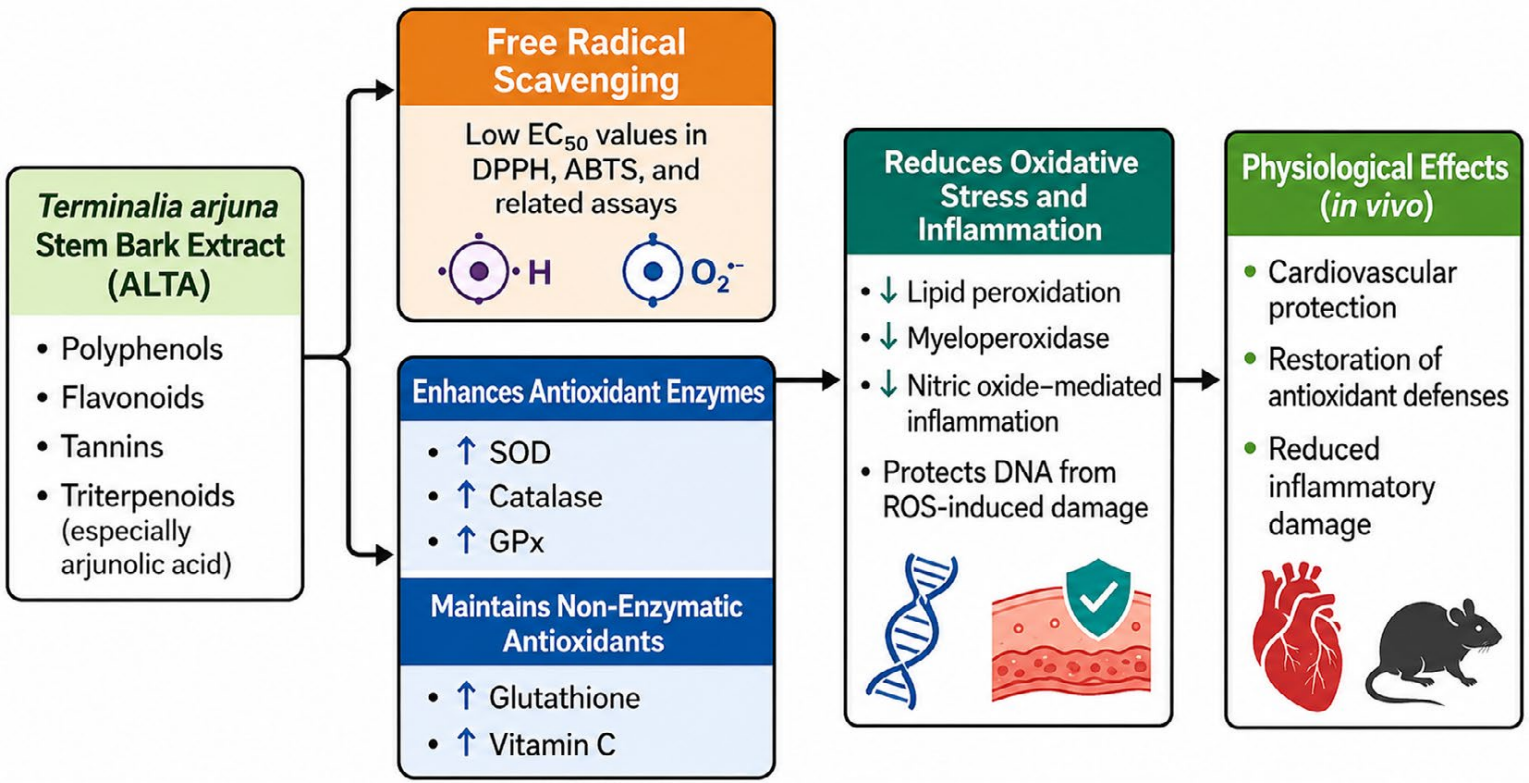
Antioxidant properties of *Terminalia arjuna*.

*Terminalia arjuna* bark powder has similar protective effects in vivo with supplementation. It has shown to decrease the negative products of oxidative stress (malondialdehyde, nitric oxide, and myeloperoxidase) and increase the normal level of renal functioning by reducing the high levels of creatinine and uric acid in the blood of animal models of induced oxidative stress. It was also found that the extract reinstated antioxidant enzyme activities, restored redox balance, and alleviated structural damage in kidney tissue. These results indicate the strong antioxidative and reno‐protective effects of *T. arjuna*, which justifies its traditional use and indicates that it has therapeutic potential in oxidative stress‐induced kidney diseases (Haque et al. 2025; Kanthe et al. 2022). Figure 6 shows the antioxidant properties of *T. arjuna*.

### 2.8 Anticancer Properties

Cancer is the malignant form, which proliferates to the other organs of the body. It has been estimated that ~20 million newly diagnosed cases of cancers are reported globally throughout the year, with ~9.7 million mortality rates (Raza et al. 2025). In recent research, there has been notable anti‐cancer activity of *T. arjuna* due to its significant cytotoxic and pro‐apoptotic activity in different types of cancer. Furthermore, *T. arjuna* alcoholic bark extracts showed a high cytotoxic and cytostatic effect on human T‐lymphoblastic (Jurkat) leukemia cells. It induced apoptosis and necrosis and led to the stagnation of the cell cycle in the G2/M stage, which is a practical way to stop the multiplication of cancer cells (Greco et al. 2021).

Among several human cancer cell lines, including oral (KB), liver (HepG‐1 and WRL‐68), and ovarian (PA‐1) cells, arjunic acid is the most effective chemical from *T. arjuna* bark extracts. Furthermore, 2‐O‐palmitoyl arjunic acid and other semi‐synthetic ester derivatives of arjunic acid showed double the cytotoxic action of the parent chemical, indicating that *T. arjuna* triterpenoids have structure‐dependent activity in cancer cells (Saxena et al. 2007). Other triterpenoids like arjunolic acid amide derivatives (mainly TA‐1,4‐Bip) have been studied for their anti‐cancer role recently. The compound has been shown to induce cell cycle arrest at the G_0_/G_1_ phase in cancer cells and greatly increases intracellular reactive oxygen species (ROS) along with activating apoptosis to reduce cell viability (Bhujel et al. 2025).

Moreover, in vivo studies reported that the hydroalcoholic extract of *T. arjuna* demonstrated a substantial effect against dibutyltin dichloride (DBTC)‐induced (pancreatic cancer in animal models). Both 250 and 500 mg/kg doses for 28 days had a noteworthy reduction in serum levels of α‐amylase, lipase, and glucose (key biochemical markers in pancreatic inflammation and tumor). These findings suggest that *T. arjuna*'s rich phytochemical profile contributed to mitigation of pancreatic cell proliferation, making it a botanical candidate for pancreatic cancer management (Harshitha and Gorla 2021).

More recently, Adhikary and Basak (2024) investigated in silico virtual screening of *T. arjuna* bark compounds to identify multi‐target compounds against proteins implicated in lung cancer pathogenesis. Their findings reported that *T. arjuna* phytoconstituents had a high binding affinity as compared to standard anti‐cancer drugs and these phytochemicals proved to be effective against ALK2, ALK5, DDR2, BRAF, KRAS, VEGFR1 and VEGFR2 mediated lung cancer, however these had limited efficacy against Bcl‐2, NCAM, RET, MET and ROS1 receptors. Nano‐formulated *T. arjuna* bark extracts, chiefly the bark‐derived triterpenoids and polyphenols show strong activity against breast cancer cellular targets. *Terminalia arjuna* bioactives may disrupt breast cancer cell regulatory mechanisms by modulating pathways involved in tumor suppression (BRCA1), immune response (β‐Defensin 6) and cell proliferation control (neurofibromin), along with this the targeted nano‐delivery of phytochemicals can enhance the anticancer potential (Padmavathy et al. 2024). Additionally, the petroleum ether bark extract of *T. arjuna* in vitro cytotoxic assays against human liver (HEP2) and colon (HT29) cancer cell lines suggest that at 100 μg/mL, the extract exhibits significant growth reduction, up to 82% in HEP2 and 84% in HT29 cells compared to the anticancer drug Mitomycin‐C. This activity was quantified by Sulforhodamine B (SRB) assay that measures total cellular protein to assess cell viability (Singh et al. 2017).

Furthermore, in combined in vitro and in vivo investigations, *T. arjuna* bark extract has been found to have antimutagenic effects against aflatoxin B_1_ (AFB_1_) that is a well‐known environmental genotoxin. Aqueous extracts (75–200 μg/mL) substantially decreased chromosomal aberrations and sister chromatid exchanges, while improving the replication index from 1.33 to 1.55. In vivo experiments in albino mice further confirmed the extract's protective effect: pretreatment with oral doses of 50–350 mg/kg body weight reduced AFB₁‐induced clastogenicity, with aberrant cell frequencies dropping from 429 (positive control) to 141 at the highest extract dose after 32 h, demonstrating up to ~55% inhibition of chromosomal damage. The plant's rich profile of antioxidant polyphenols, flavonoids, and triterpenoids, which support genomic integrity under toxic stress, is probably responsible for these protective effects (Ahmad, Ahmad, et al. 2014; Ahmad, Butt, et al. 2014).

Liu et al. (2019) investigated the anticancer activity of *T. arjuna* bark extracts in lung (A549, H1975) and breast cancer models, including Wnt‐transgenic mice with mammary tumors. At 50 μg/mL, both aqueous and ethanol extracts inhibited cancer cell proliferation within 24 h, with minimal impact on normal lung fibroblasts (CCD‐19Lu). The ethanol extract was slightly more potent, and both extracts induced apoptosis, evidenced by cytoplasmic vacuolization and mitochondrial membrane depolarization, particularly in H1975 cells. Moreover, filtration assays indicated that active compounds were > 10 kDa, suggesting involvement of high‐molecular‐weight phytoconstituents. In vivo, oral administration of the aqueous extract (15 mg/kg) significantly reduced tumor size, with no recurrence observed for up to 1 month post‐treatment.

Collectively, the studies reviewed suggest that *T. arjuna* contains a diverse set of compounds capable of acting against cancer through several mechanisms, including growth inhibition, induction of apoptosis, and protection against DNA damage.

### 2.9 Anti‐Inflammatory Properties

Inflammation is the key anomaly to all chronic disease conditions, and it is the main cause of disease progression. Natural compounds are being highly explored for their anti‐inflammatory effects. *Terminalia arjuna* which has long been used in the Ayurvedic medicine system in cardioprotective mechanisms, has also proven to be active in inflammatory diseases with experiments showing sound reduction of inflammation in various disease conditions.

*Terminalia arjuna* bark aqueous and ethanolic extracts exhibit noteworthy anti‐inflammatory activity in egg albumin denaturation assay. Both extracts showed strong inhibitory effect towards protein denaturation across tested volumes of 10–50 μL, with the aqueous extract showing higher activity than the ethanol extract even at low concentrations (10–20 μL) (Vijayalakshmi et al. 2023). Mechanistically, the inhibition of protein denaturation is attributed to strong suppression of inflammatory mediators and stabilizing effect of *T. arjuna* polyphenols. The anti‐inflammatory potential of *T. arjuna* is mainly due to a rich composition of phytochemicals like arjunic acid, arjunjenin, arjunolic acid, terminic acid, flavonoids, and tannins (Jaiswal et al. 2021). The bark extract inhibits pro‐inflammatory mediators like prostaglandins, stabilizes cytokine levels, and scavenges free radicals to reduce oxidative stress, among other biological mechanisms. These exercises lessen inflammation without having any negative effects side symptoms frequently linked to corticosteroids and NSAIDs (Dube et al. 2017).

More recently, Verma, Vyas, and Nama (2025) developed an oral suspension containing 500 mg of *T. arjuna* bark extract per 5 mL, targeting pediatric and geriatric populations. The anti‐inflammatory efficacy of this formulation was evaluated using the carrageenan‐induced paw edema model in Wistar rats, a widely accepted in vivo assay for anti‐inflammatory activity. Administration of the *T. arjuna* suspension produced a significant reduction in paw swelling, comparable to the standard drug diclofenac sodium. The anti‐inflammatory effect was observable within the first few hours and persisted throughout the observation period, indicating both rapid onset and prolonged duration of action. This showed that *T. arjuna* bark extract can effectively exert anti‐inflammatory effect similar to most medications. Similarly, in vivo investigation of ethanolic bark extract at three dose levels, 250, 500, and 1000 mg/kg body weight demonstrated a dose‐dependent response. It was noted that higher dose produced a more significant reduction (63.79%) in inflammation which was closer to the effects of reference drug diclofenac sodium (66.19%), confirming anti‐inflammatory activity of *T. arjuna* bark extracts (Tahsin et al. 2021).

In a comparative study using the same edema model, two formulations were evaluated for anti‐inflammatory effects of *T. arjuna*: a hydrolytic extract (HA) and a traditional Ayurvedic preparation called Arjuna Ksheera Paka (AKP), a decoction prepared by boiling bark powder in milk and water (Timilsina et al. 2024). Both extracts were tested in Swiss albino mice at oral doses of 200, 400, and 800 mg/kg. While the HA extract showed stronger antioxidant activity in vitro, AKP demonstrated superior in vivo anti‐inflammatory activity, especially in the late phase of inflammation (48–96 h). At the maximum dosage of 800 mg/kg, AKP exceeded HA in lowering inflammation over time and achieved up to 90.73% reduction of paw swelling, almost matching the conventional medication aspirin. The enhanced efficacy of AKP is attributed to the presence of milk solids, which are believed to enhance the bioavailability and sustain the release of active phytochemicals. Mechanistically, AKP appeared more effective in controlling late‐phase inflammatory mediators, such as prostaglandins (via COX‐2 inhibition), while HA had a stronger impact in the early phase through modulation of COX‐1 and nitric oxide pathways (Dube et al. 2017).

Various studies have demonstrated that extracts from *T. arjuna* bark can inhibit the production of nitric oxide in lipopolysaccharide (LPS)‐stimulated macrophages, causing a significant decrease in inflammatory responses (Rajaram et al. 2024). Abo‐Elghiet et al. (2022) compared methanol extracts from *T. arjuna* leaves, fruits, and bark for anti‐inflammatory effect using human red blood cell (HRBC) membrane stabilization assay. At the low concentration of 5 μg/mL, the leaves extract demonstrated an outstanding protection of HRBCs against lysis, reaching 95.71% of membranes stabilization, which is much higher than those of the fruit extract, that performed 98.22% stabilization at 10 μg/mL. These were strongly associated with increased phenolics, flavonoids, and tannins concentration in the leaf and fruit extracts than in the bark. These findings indicate that the non‐bark components of *T. arjuna* could provide even more potential anti‐inflammatory effects because of their detailed phytochemical compositions, although in vivo verification is needed.

**FIGURE 7:**
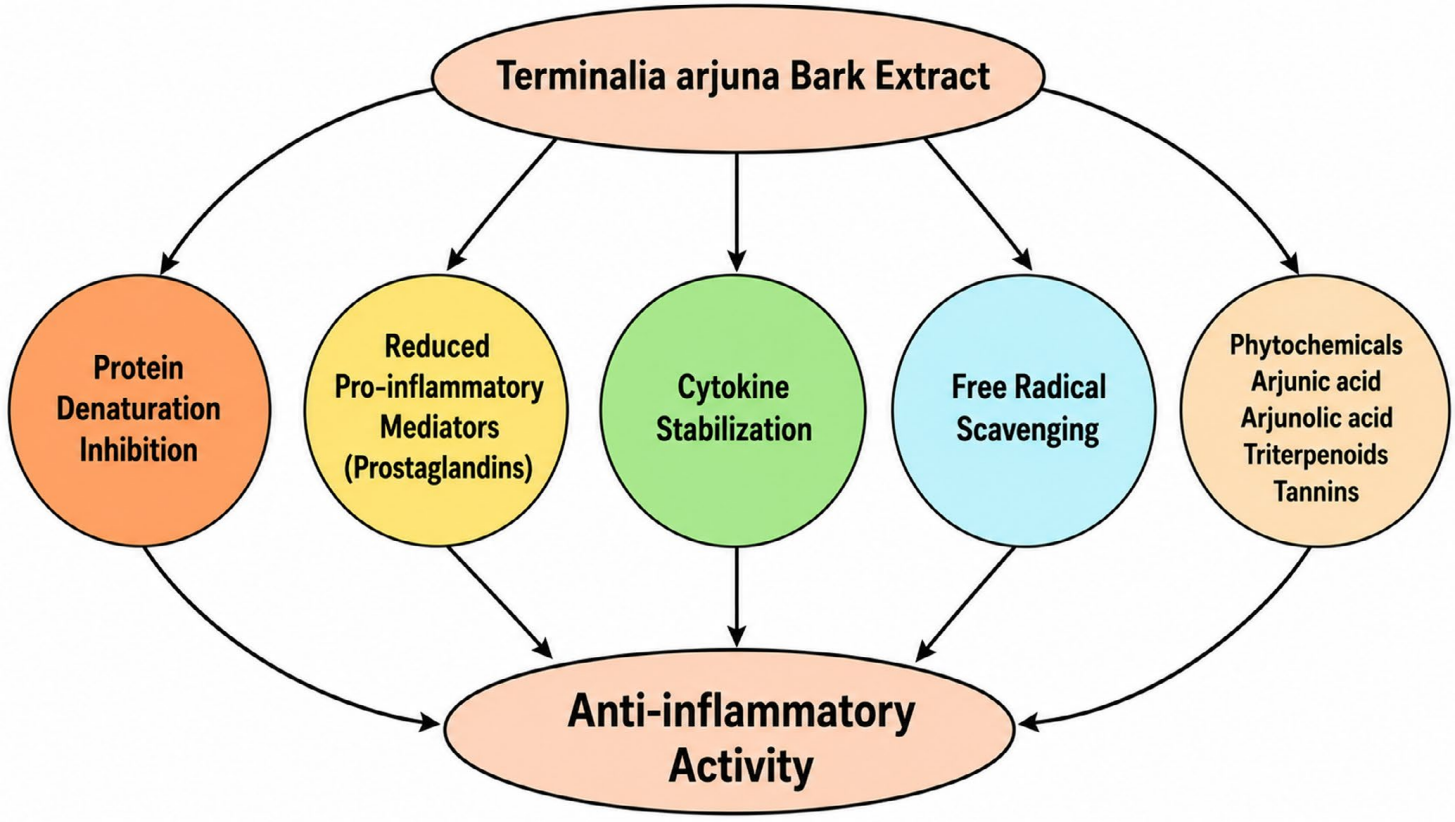
Anti‐inflammatory properties of *Terminalia arjuna*.

Furthermore, in acute and chronic inflammation models, *T. arjuna* leaf extracts significantly reduced inflammation. At oral doses of 200 and 400 mg/kg the methylene chloride‐methanol leaf (MMTL) extract of *T. arjuna* achieved 38.0% and 64.6% decrease in inflammation respectively. In chronic inflammation, the 400 mg/kg dose resulted in a decrease in weight of granuloma tissue (as investigated in the cotton pellet‐induced granuloma study model) of almost 30%, as compared to the control group (the dose reduced granuloma weight to ~73.7 mg compared to ~115 mg in the control), whereas the lower dose had less significant results. Hematological and biochemical results further demonstrated that MMTL caused a decrease in the number of white blood cells and platelets, and an increase in hemoglobin and the number of red blood cells indicating an anti‐inflammatory full‐body effect of MMTL. Furthermore, MMTL therapy reduced oxidative stress parameters, nitrite and lipid peroxidation, and increased cellular antioxidant enzymes, such as SOD (superoxide dismutase), GSH (glutathione), and GP (glutathione peroxidase) in liver and exudate tissues (Sarma et al. 2019). The anti‐inflammatory properties of *T. arjuna* are represented below in Figure 7.

### 2.10 Cardioprotective Activity

*Terminalia arjuna* has a long history in Ayurveda as a heart tonic, and modern research strongly supports its cardioprotective role. The bark extracts have an improving effect on cardiac output and coronary blood flow, while also protecting against ischemia and heart failure due to its ability to boost antioxidant enzymes like SOD, catalase, and GPx. Additionally, *T. arjuna* extracts also improve vascular function as it reduces vascular resistance by nitric oxide and cGMP signaling. Apart from this, it regulates survival pathways such as PI3K/Akt, PPAR‐γ, and LXR‐α (Upadhyay et al. 2024; Khaliq and Fahim 2018). Some animal studies demonstrate that there are advantages in diabetic heart damage by lowering blood glucose, HbA1c, and cardiac biomarkers including CPK‐MB and hs‐CRP, as well as normalizing DPP IV activity, decreasing hypertrophy, and maintaining myocardial structure (Mohanty, Borde, Kumar, and Maheshwari 2019; Mohanty, Borde, and Maheshwari 2019).

**FIGURE 8:**
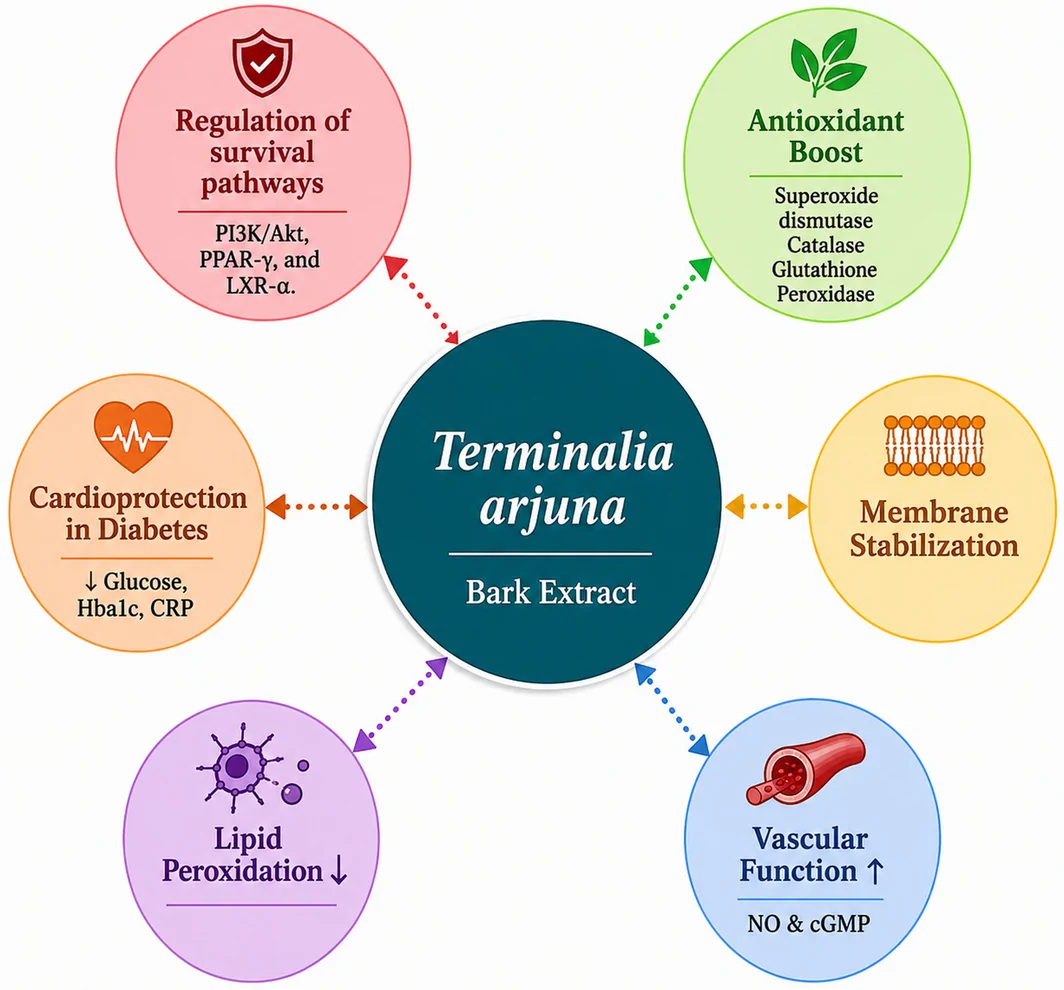
Cardioprotective properties of *Terminalia arjuna*.

Apart from this, *T. arjuna* exhibits potent lipid‐lowering properties in addition to its anti‐ischemic and antidiabetic properties. Recent studies suggest that *T. arjuna* bark extract was as effective as atorvastatin in lowering cholesterol, triglycerides, and LDL in high‐fat diet rat models while also restoring HDL, enhancing antioxidant defense, and protecting heart tissue (Palanivelu et al. 2023). By blocking thrombin and ACE, lowering apoptotic indicators, and protecting cardiomyocytes from oxidative stress, standardized formulations such as Cardiboost (1% arjunolic acid) further demonstrated protective effects (Gouthamchandra et al. 2024). Studies on bark powder also revealed elevated antioxidant genes including Nrf‐2, HO‐1, and HO‐2 and decreased oxidative stress indicators like MDA, NO, MPO, and APOP, underscoring its genomic significance in heart protection (Alimullah et al. 2024). All of these results support *T. arjuna's* potential as a multi‐target cardioprotective drug against ischemia, diabetes‐related heart damage, oxidative stress and hyperlipidemia. The cardioprotective properties of *T. arjuna* are shown below in Figure 8.

### 2.11 Antimicrobial Effects

*Terminalia arjuna* exhibits antimicrobial effects that extend its therapeutic potential into the management of infectious diseases. Polar extracts of *T. arjuna* (aqueous, methanolic, ethanolic, acetone) have always demonstrated that they are strong antibacterials against such pathogens as *E. coli* , *Pseudomonas aeruginosa* , *Acinetobacter baumannii* , *Enterococcus faecalis* , and *Staphylococcus aureus* , with the MIC values ranging from milligrams to micrograms per milliliters (Debnath et al. 2013; Abo‐Elghiet et al. 2022; Vijayalakshmi et al. 2023). Moreover, the protective effects of *T. arjuna* bark and leaf extracts against ear and oral infections are also shown to be comparable and sometimes even better than standard antibiotics and mouthwashes (Vashistt 2022; Dandu et al. 2020). Most recent antimicrobial uses of *T. arjuna* include its incorporation into dental cement to eliminate *Streptococcus mutans* without affecting the strength of the material (Devi et al. 2024) and into food systems, where ethanolic extracts extended the shelf life of goat meat by reducing the level of microbes, slowing lipid oxidation, and enhancing the taste and texture (Singh et al. 2025). Mechanistically, the antimicrobial activity of *T. arjuna* is primarily attributed to the ability of its tannins and polyphenols to disrupt bacterial cell membrane integrity, inhibit essential microbial enzymes, and chelate metal ions required for bacterial survival, while flavonoids such as luteolin additionally contribute through inhibition of bacterial DNA gyrase and suppression of cell wall synthesis (Zhang et al. 2025; Olchowik‐Grabarek et al. 2022).

Extracts of *T. arjuna* leaves tend to have a slightly better antimicrobial power compared to bark extracts because of the larger concentration of phenolic compounds, along with cytotoxicity found in cell models (Jagtap et al. 2024). Besides human health, *T. arjuna* has been reported to be useful in aquaculture by destroying bacteria, fungi, and parasites. It also works by eliminating bacterial and fungal infections in fish through water treatment and dietary supplementation along with improving immunity without any toxic effects (Meena et al. 2025). Collectively, the findings show that *T. arjuna* is a multifunctional natural antimicrobial, which can be used in clinical oral care or food preservation and aquaculture, and therefore makes it a candidate used for the reduction of synthetic antimicrobials use.

### 2.12 Hepatoprotective Effects

*Terminalia arjuna* extracts show strong hepatoprotective effects across multiple liver injury models. For instance, purified arjunolic acid reduced triglyceride buildup in HepG2 cells by 66% and lowered ALT/AST leakage by up to 61%. In vivo it improved liver enzymes and reduced steatosis through modulation of PPARα, FXRα, and PPARγ signaling (Toppo et al. 2018). Additionally, ethanolic bark extracts protected against isoniazid‐ and acetaminophen‐induced liver damage by normalizing AST/ALT levels, boosting antioxidant enzymes (SOD, catalase, glutathione), and suppressing caspase‐9 and IL‐1β pathways, confirming their role in reducing oxidative stress, inflammation, and apoptosis (Iqram et al. 2025a, 2025b; Kannappan et al. 2020). At the molecular level, the hepatoprotective effects of *T. arjuna* are orchestrated through multiple interconnected pathways. Arjunolic acid and polyphenols activate the Nrf2/HO‐1 signaling axis, which upregulates cytoprotective and antioxidant genes, reducing hepatocellular oxidative damage. Simultaneously, suppression of the NF‐κB pathway by *T. arjuna* bioactives downregulates pro‐inflammatory mediators including IL‐1β and TNF‐α, limiting inflammatory infiltration of hepatic tissue. At the metabolic level, modulation of PPARα, FXRα and PPARγ nuclear receptors by arjunolic acid regulates lipid metabolism, reduces triglyceride accumulation, and improves hepatic steatosis. Furthermore, inhibition of caspase‐3 and caspase‐9 dependent apoptotic signaling preserves hepatocyte integrity under toxic and oxidative stress conditions, collectively contributing to the restoration of normal liver architecture and function (Sherif 2021).

Further studies confirm benefits in metabolic and toxic liver injury. High‐dose ethanolic extracts improved liver enzyme profiles and favorably modulated HDL and LDL in hyperlipidemic rats, without harming renal function (Rahmat et al. 2024). Earlier work also showed that methanolic extracts reversed cadmium‐induced hepatic toxicity by lowering ALT, AST, ALP, and lipid peroxidation (MDA), while restoring antioxidant defenses (Jain and Singh 2023). Taken together, these findings highlight *T. arjuna* as a potent hepatoprotective agent, acting through antioxidant, anti‐inflammatory, and metabolic regulatory pathways.

### 2.13 Gastroprotective Effects

It is mainly because of the bioactive phytoconstituents (including polyphenols, tannins, flavonoids, triterpenoids (arjunolic acid), and ellagic acid) that *T. arjuna* has pharmacological effects, including antioxidant, anti‐inflammatory, anti‐ulcerative, and mucosal protective properties (Kapoor et al. 2014; Amalraj and Gopi 2017). This is mainly because, at the mucosal defense level, *T. arjuna* extracts strengthen the gastric mucosal barrier by increasing adherent mucus content, elevating protein‐bound carbohydrate complexes (hexose, hexosamine, sialic acid, and fucose) that serve as mucin surrogates and raising non‐protein sulfhydryl (NP‐SH) levels which are critical for mucosal integrity. Simultaneously, *T. arjuna* reduces gastric acid secretion by lowering free and total acidity, pepsin concentration, and acid output, thereby reducing the ulcerogenic burden on the mucosa. At the cellular level, its polyphenols and triterpenoids exert potent anti‐lipid peroxidative activity by scavenging free radicals and restoring enzymatic antioxidants including SOD, CAT, GPx, and GST, while reducing MPO activity and LPO levels in gastric tissue. Furthermore, inhibition of NF‐κB activation by *T. arjuna* suppresses downstream inflammatory cytokine production including TNF‐α and IL‐6, reducing mucosal inflammation. Collectively, these cytoprotective, antioxidant, and anti‐inflammatory mechanisms work in concert to maintain membrane integrity and accelerate mucosal healing (Devi et al. 2007; Mukherjee 2020; Cota et al. 2019).

Peptic ulcers are one of the most studied gastrointestinal disorders and several preclinical studies have shown that the *T. arjuna* (bark) extract can be used to protect the gastrointestinal mucosa. A multi‐model study of *T. arjuna* extract (TAE) using rats showed that a methanolic extract of the TA bark had a strong anti‐ulcer and ulcer‐healing efficacy on 80% ethanol, diclofenac sodium, and dexamethasone injury. It was dose‐dependent and the effective dose of 100 mg/kg (ethanol model), 400 mg/kg (diclofenac model), and 200 mg/kg (dexamethasone model) was applied. TAE enhanced the concentrations of the gastric protein‐bound carbohydrates complexes (mucin surrogates), reduced lipid peroxidation (LPO), and enhanced SOD and CAT along with improving histology, facilitated maintenance of epithelial integrity, and reduced hemorrhagic lesions (Devi et al. 2007). The same results were observed by Mishra et al. (2021), who showed that the *T. arjuna* ethanolic extracts possessed a highly protective effect against pylorus ligated ulcers, demonstrating broad‐spectrum gastroprotective activity.

The gastroprotective effects of *T. arjuna* extend beyond peptic ulcer models. Its role in attenuating colitis‐associated mucosal damage, reducing pro‐inflammatory cytokines, restoring antioxidant enzyme activity, and preserving epithelial barrier integrity has been discussed in detail in the preceding section on its role in ulcerative colitis.

### 2.14 Antidiabetic Effects

Diabetes mellitus and, in particular, Type 2 diabetes is a chronic hyperglycemic, insulin‐resistant, dysfunctional and oxidatively stressed cell condition (Accili et al. 2025, Sultan et al. 2014). The evidence of the antidiabetic properties of the *T. arjuna* strongly suggests that the plant might improve insulin secretion, enzyme activities, antioxidant activity, and lipid metabolism (Tahir et al. 2025). *Terminalia arjuna* possesses a potent antidiabetic effect in many respects and some of its effects involve stimulation of insulin release, stimulation of glucose uptake, inhibition of enzymes, antioxidant effect, and control of lipid metabolism. The extracts of bark encouraged calcium dependent insulin release in vitro, improved glucose absorption in adipocytes, inhibited starches and glycated protein digestions. These were validated in animal research: oral administration of extracts of bark or the fruit lowered fasting glycaemia, postprandial glycaemia, reduced glycaemia as indicated by HbA1c, increased insulin, decreased DPP IV and preserved β‐cell functions and incretin signaling (Mohanty, Borde, Kumar, and Maheshwari 2019; Mohanty, Borde, and Maheshwari 2019). Moreover, the treated animals also had improved lipid profile, reduced oxidative stress (decreased MDA), improved antioxidant enzymes (SOD, catalase, glutathione), and preserved pancreatic structure of the islet.

Another clinical study with Type 2 diabetes patients showed reduced fasting glucose and HbA1c (0.1%) and lipid parameter improvement and no adverse hepatic and renal side effects (Prakash et al. 2024). A second study about Kaand Twak Churna indicated a symptomatic benefit, albeit with a decrease in glucose levels and a similar improvement in glycemic control, while an Ayurvedic tablet preparation (Gutika) standardized with *T. arjuna* extract showed an increase in glucose tolerance, insulin secretion, lipid status, and antioxidant status (Engle 2025). Generally, it has been pointed out that *T. arjuna* possesses multidimensional antidiabetic effects which are mediated by its effect on β‐cell protection, incretin modulation, oxidative stress, and reduction of metabolic balance which make it a good candidate drug to be employed as adjunct therapy in diabetes Type 2.

### 2.15 Renal‐Protective Effects

Recent studies in the last 10 years have shown that *T. arjuna* is a potential nephroprotective botanical, which can ameliorate different types of kidney damage using antioxidant, anti‐inflammatory, anti‐lithiatic, and cytoprotective effects. Its efficacy is supported by studies both in rodent models and in vitro models of chemically induced nephrotoxicity and urolithiasis (Mahajan 2024). At the molecular level, the renoprotective effects of *T. arjuna* are primarily mediated by arjunolic acid, which attenuates oxidative stress markers, downregulates renal expressions of fibrotic markers (TGF‐β), inflammatory markers (NF‐κB), and kidney injury markers (Kim‐1), while simultaneously upregulating antiapoptotic signaling (Bcl‐2), collectively preserving renal tubular integrity and function (Elsherbiny et al. 2016).

Das et al. (2016) used a traditional experimental design to observe the influences of methanol fraction extracts of *T. arjuna* (bark) on male Wistar rats, 15 days of water deprivation. Dehydrated rats showed an increase in blood urea nitrogen (BUN), creatinine, and oxidative markers like MDA and reduced renal antioxidant enzymes (SOD, catalase, peroxidase). Oral delivery of oral bark methanol fractions at 10–80 mg/kg/day restored baseline values of BUN and creatinine and dose dependently restored oxidative stress biomarkers and enzyme activities and did not alter serum transaminases, indicating that it was renal safe and effective in preventing uremia and oxidative kidney injury. In addition, other recent studies also indicated that *T. arjuna* bark powder supplementation reduced serum creatinine and uric acid levels much in mice with cardiorenal injury induced by isoproterenol, which indicated systemic antioxidant and anti‐inflammatory signaling that also maintained renal function (Haque et al. 2025).

In their study, Rai et al. (2020) tested the anti‐urolithiatic properties of *T. arjuna* in male rats inflicted with kidney stones by administering 0.75% ethylene glycol + 1% ammonium chloride. The ethanolic bark extract (200 and 400 mg/kg) was found to be an effective anti‐stone and antioxidant by reducing urinary calcium, oxalate, phosphate, BUN, and serum creatinine; restoring reduced glutathione and catalase levels; reducing MDA; and rehabilitating renal histology in prevention and cure (significant in both preventive and curative regimens). In addition, methanolic bark *T. arjuna* containing high levels of tannins and polyphenols at 10–80 mg/kg/day improved increased BUN, creatinine, oxidative stress, and retained histological kidney in dehydrated rats—an indication of uremia and oxidative injury to the kidney (Rai et al. 2023). Prior, Raj et al. (2012) demonstrated that aqueous extract of the bark at the concentration of 50 mg/kg safeguards mice against the CCl4 induced oxidative injury in the kidney tissue. Seven‐day pretreatment before CCl4 challenge reestablished antioxidant enzyme (SOD, CAT, GST), decreased MDA, and maintained GSH in renal homogenates showing strong antioxidant defense responses of *T. arjuna*.

### 2.16 Wound Healing

*Terminalia arjuna* exhibits strong wound‐healing potential which supports its traditional use in medicine (Verma, Vyas, and Nama 2025). Its flavonoids, tannins, polyphenols, and triterpenoids act through anti‐inflammatory, antimicrobial, and tissue‐regenerative mechanisms. Studies show that ethanolic bark extracts accelerate wound closure, re‐epithelialization, collagen synthesis, and tensile strength while reducing inflammation (Rajaram et al. 2024).

Zinc oxide nanoparticles synthesized with *T. arjuna* further enhanced healing by boosting epithelialization, granulation tissue formation, collagen deposition, lowering oxidative stress, and preventing infections from pathogens like *Staphylococcus aureus* and *Pseudomonas aeruginosa* (Tahir et al. 2025). Reviews also highlight its tannin‐rich profile for antimicrobial and astringent effects that limit bacterial growth, regulate inflammation, and support faster tissue repair, positioning *T. arjuna* as a promising natural wound‐care agent (Sharma et al. 2021).

### 2.17 Adaptogenic and Stress‐Reducing Effects

*Terminalia arjuna* is increasingly recognized as an adaptogenic plant which can protect against physiological disruption caused by stress. Prolonged stress causes an increase in cortisol, oxidative injuries, defective body defense, and psychological disorders. According to Parmar et al. (2006), *T. arjuna* aqueous bark extracts of 250 and 500 mg/kg reversed stress‐induced elevations in serum cortisol and prevented behavioral changes in immobilization stressed rats. In addition, the treatment reduced the oxidative stress biomarkers to a significant degree, and it recovered the antioxidant enzyme activity, such as superoxide dismutase and catalase. In line with these observations, chronic intake of *T. arjuna* bark extract had a substantial effect to reduce anxiety‐like behaviors caused by chronic stress in rodents. The animals that were given the extract exhibited some normalization in corticosterone, decreased gastric lesions, better antioxidant condition, and tremendous enhancement in open‐field behavioral tests, thus validating its adaptogenic and stress‐relieving virtues (Tahsin et al. 2021).

More recently, a comprehensive review by has identified the implication of polyphenol‐containing adaptogens like *T. arjuna* in the stress management process. The bioactive constituents of it, especially flavonoids and tannins, according to this review, assist in homeostasis through safeguarding neuronal cells against oxidative stress related to stress, regulating neurotransmitter systems, and mitigating endocrine reactions, having a beneficial effect on psychological and physiological resistance to persistent stressors.

### 2.18 Food Applications

*T. arjuna* is an excellent source of phytochemicals with therapeutic multimodality, which has enabled its use in functional foods and supplements. Meena et al. (2024) demonstrated the positive results of dietary supplementation on freshwater fish *Labeo rohita* with *T. arjuna* bark powder in aquaculture. Supplementation of fish diets with graded amounts of bark powder (1, 10, and 15 g/kg) over a 90‐day period beneficially altered fish growth performance, hematological parameters, immune status, and resistance to bacterial pathogens, including *Aeromonas hydrophila* and *Edwardsiella tarda* . The histological analysis revealed significant liver tissue protection and improved gut condition, which is why *T. arjuna* is a promising feed additive to supplement aquaculture with an improved productive effect (Meena et al. 2024).

Besides, recent phytochemical studies support the possibility of *T. arjuna* extracts application in human functional foods. The existence of potent antioxidants like ellagic acid, gallic acid, and arjunolic acid makes *T. arjuna* a very interesting natural preservative and functional ingredient which could potentially increase nutritional content, improve metabolic fitness, and provide antioxidant properties when used in nutraceuticals.

### 2.19 Dosage and Safety Data

The development of *T. arjuna* as a therapeutic agent is based on determining the dosage and safety of this drug in the management of UC. Although different sources of preclinical and traditional medicine indicate the use of *T. arjuna* in digestive and cardiovascular diseases, there is still no clear agreement on the safety and efficacy of *T. arjuna* in effective dosing among human UC groups. In Ayurvedic medicine, powdered formulations of the bark are usually taken in 500 mg to 2 g doses twice daily. The standardized extracts of *T. arjuna* have been used in clinical studies investigating it in non‐UC conditions, including chronic gastritis, hyperlipidemia, and cardiovascular ailments, with doses of between 250 and 1000 mg once to twice daily (Mohanty, Borde, Kumar, and Maheshwari 2019; Mohanty, Borde, and Maheshwari 2019). Nonetheless, such doses should be extrapolated to UC patients with caution, in particular in light of the intricate and chronic inflammatory microenvironment of UC.

In preclinical colitis models, aqueous or hydroalcoholic extracts of *T. arjuna* administered at doses of 100–500 mg/kg in rodents have demonstrated consistent anti‐inflammatory and antioxidant effects. For instance, in DSS‐induced colitis, a 200 mg/kg dose reduced colon shortening, ulceration, and histological inflammation, correlating with reduced TNF‐α, IL‐1β, and IL‐6 levels (Khan et al. 2022). On the same note, arjunolic acid isolated from *T. arjuna* demonstrated strong effects at 50 mg/kg in TNBS‐induced models with significant indices of oxidative stress reduction and glutathione restoration (Sharma 2022). Although these results are promising, the animal‐to‐human dose conversion should be meticulously scaled with the help of the common allometric scaling parameters. A 200 mg/kg dose in rats through the FDA guidelines correlates to 32 mg/kg in humans which translates to about 2.2 g in an adult of 70 kg. Thus, the rational and possibly effective range of human‐equivalent doses of 1 g to 2.5 g/day of crude bark powder or standardized extract is within the rational range.

Animal model safety testing has mostly indicated a high margin of safety to *T. arjuna* extracts. In acute toxicity studies, no adverse effects or mortality were reported at doses up to 2000 mg/kg in rats. Up to 90‐day sub‐chronic administration also did not result in any significant changes in hepatic or renal biomarkers, hematological monitoring, or histopathological observations (Patel et al. 2025). Even the highest dosage of 1000 mg/kg/day showed no toxicological deviation in gastrointestinal tissues, which further proved the safety of the high doses of *T. arjuna*. Nonetheless, some of the preparations, including alcoholic extracts, can also differ in safety profile with regard to the solvent used in extracting active constituents, which can alter phytochemical concentration and bioavailability.

Human safety data, though limited, supports a favorable profile. The results of a clinical study involving 80 patients with chronic gastritis who were given a standardized *T. arjuna* extract (500 mg twice a day) throughout 8 weeks showed a significant reduction in symptoms without any significant adverse events or alteration of liver and kidney functions (Kapoor et al. 2014). Similarly, clinical studies on cardiac patients taking *T. arjuna* bark powder (maximum 3 g/day) during a period of more than 6 months demonstrated marked increases in ejection fraction and lipid profiles without hepatotoxicity and nephrotoxicity (Dwivedi and Chopra 2014). Nevertheless, the results might not be directly applicable to UC patients, where the long‐term immune modulation and gut microbiota alterations might affect safety.

### 2.20 Future Aspects

*Terminalia arjuna* is used in UC management, there are still certain gaps which need to be addressd. Absences of standard formulations and dosage regimes are one of the greatest concerns. Most of the studies that have been done have utilized crude extracts without precise quantification of phytochemicals. This results in inconsistency of outcomes and interference with the establishment of an appropriate dose. To be regarded as a viable therapeutic agent, standardized extracts confirmed by chromatographic analysis such as HPLC or LC–MS/MS will have to be accepted to provide consistency and dose fidelity. Till now, no randomized controlled trials have been done to study its safety or effectiveness in UC patients. Though clinical trials in gastritis and irritable bowel syndrome have demonstrated efficacy with little adverse events (Kapoor et al. 2014), these cannot be directly applied to UC because of the chronicity and the complexity of immunological response to the disease. Future studies should involve serious trials on the well‐characterized *T. arjuna* preparations, with outcome measures like mucosal healing, disease activity measures, biomarkers of inflammation, and quality‐of‐life parameters. *Terminalia arjuna* should also be tried as an adjunct against conventional therapy like mesalamine or corticosteroids. Besides the clinical approval, more mechanistic understanding is needed to clarify the loci altered by *T. arjuna*. Although the studies indicate the modulation of NF‐κB and Nrf2 in other inflammatory settings, there are still limited studies that directly indicate such effects in the UC models. Research on its immunomodulatory actions can be improved by investigations on the organoid colonic structures, transcriptomic analyses, and molecular docking studies. Also, the impact of *T. arjuna* on the diversity of gut microbiota that seems to be promising with preclinical colitis models ought to be confirmed using 16S rRNA sequencing in human samples. Lastly, long‐term safety data and pharmacokinetic profiling are essential before broad therapeutic use. Although animal studies report a high safety margin, chronic toxicity data in humans especially in combination with immunosuppressants, is lacking. Herb‐drug interaction studies and ADME profiling are critical to prevent unintended consequences when used alongside standard UC medications.

## 3 Conclusion

*Terminalia arjuna* has predominant significance in healthcare sectors, particularly involved in the management of ulcerative colitis by alleviating its etiology, pathogenesis, and progression. Moreover, its antioxidant, anti‐inflammatory, mucosal protective, and immunomodulatory properties result in reducing the severity of UC in experimental models. Prominently, bioactive compounds, like arjunolic acid, flavonoids, and polyphenols, contribute to its anti‐inflammatory actions against cytokines. Moreover, these compounds restore epithelial barrier integrity and aid in modulating gut microbiota, thereby further expanding its therapeutic relevance. Previous literature is mostly related to gastrointestinal disorders, such as gastritis and irritable bowel syndrome, but these studies are quite limited. There is a need for well‐designed clinical trials to establish optimal dosing regimens and long‐term safety in UC‐specific populations. Furthermore, herb‐drug interaction studies are essential to assess the immunosuppressive and biologic agents commonly used in UC management.

## Author Contributions

**Rameen Naeem:** writing – original draft. **Muhammad Tauseef Sultan:** conceptualization, writing – original draft. **Muhammad Usman Khalid:** validation, writing – original draft. **Muhammad Maaz:** validation, visualization. **Ayman Furqan:** data curation, formal analysis. **Laiba Tanvir:** writing – original draft. **Fabiano Travaglia:** writing – review and editing. **Matteo Bordiga:** writing – original draft. **Mutjardah Zarrish:** writing – original draft.

## Ethics Statement

The authors have nothing to report.

## Conflicts of Interest

The authors declare no conflicts of interest.

## Data Availability

The data that support the findings of this study are available on request from the corresponding author.

## References

[fsn372329-bib-0001] Abo‐Elghiet, F. , A. Abd‐elsttara , A. M. Metwaly , and A. E. I. Mohammad . 2022. “Antioxidant, Anti‐Inflammatory, and Antimicrobial Evaluation of *Terminalia arjuna* Leaves, Fruits, and Bark.” Azhar International Journal of Pharmaceutical and Medical Sciences 2, no. 2: 148–158. 10.21608/aijpms.2022.127469.1117.

[fsn372329-bib-0176] Accili, D. , Z. Deng , and Q. Liu . 2025. “Insulin Resistance in Type 2 Diabetes Mellitus.” Nature Reviews Endocrinology 21, no. 7: 413–426. 10.1038/s41574-025-01114-y 40247011

[fsn372329-bib-0002] Adhikary, T. , and P. Basak . 2024. “In Silico Approach to Investigate the Phytochemicals of *Terminalia arjuna* as Multitarget Inhibitors of Proteins Involved With Lung Cancer.” Letters in Drug Design & Discovery 21, no. 2: 329–338. 10.2174/1570180819666220913150304.

[fsn372329-bib-0003] Ahmad, M. S. , S. Ahmad , B. Gautam , M. Arshad , and M. Afzal . 2014. “ *Terminalia arjuna*, a Herbal Remedy Against Environmental Carcinogenicity: An In Vitro and In Vivo Study.” Egyptian Journal of Medical Human Genetics 15, no. 1: 61–68. https://www.ajol.info/index.php/ejhg/article/view/100847.

[fsn372329-bib-0004] Ahmad, R. S. , M. S. Butt , N. Huma , et al. 2014. “Quantitative and Qualitative Portrait of Green Tea Catechins (GTC) Through HPLC.” International Journal of Food Properties 17, no. 7: 1626–1636. 10.1080/10942912.2012.723232.

[fsn372329-bib-0007] Alimullah, M. , N. Rahman , P. Sornaker , et al. 2024. “Evaluation of *Terminalia arjuna* Bark Powder Supplementation on Isoprenaline‐Induced Oxidative Stress and Inflammation in the Heart of Long Evans Rats, Understanding the Molecular Mechanism of This Old Medicinal Plant.” Journal of Medicinal Natural Products 1, no. 1: 100004.

[fsn372329-bib-0008] Alqudah, A. , E. Qnais , O. Gammoh , et al. 2025. “Exploring Scopoletin's Therapeutic Efficacy in DSS‐Induced Ulcerative Colitis: Insights Into Inflammatory Pathways, Immune Modulation, and Microbial Dynamics.” Inflammation 48, no. 2: 575–589. 10.1007/s10753-024-02048-9. PMC12053357 38918333

[fsn372329-bib-0009] Amalraj, A. , and S. Gopi . 2017. “Medicinal Properties of *Terminalia arjuna* (Roxb.) Wight & Arn.: A Review.” Journal of Traditional and Complementary Medicine 7, no. 1: 65–78. 10.1016/j.jtcme.2016.02.003. PMC5198828 28053890

[fsn372329-bib-0011] Aneja, K. R. , C. Sharma , and R. Joshi . 2015. “Antimicrobial Activity of *Terminalia arjuna* Wight & Arn.” Ancient Science of Life 35, no. 2: 85–90. 10.1590/S1808-86942012000100011.

[fsn372329-bib-0012] Bækgaard, F. R. W. , M. D. Kjær , S. Möller , et al. 2025. “The Severity of Rectal Inflammation and Pouch Surgery Outcome in Patients With Ulcerative Colitis: A Retrospective Cohort Study.” Inflammatory Bowel Diseases 32: izaf194. 10.1093/ibd/izaf194. 40972547

[fsn372329-bib-0014] Basak, D. , M. N. Uddin , and J. Hancock . 2020. “The Role of Oxidative Stress and Its Counteractive Utility in Colorectal Cancer (CRC).” Cancers 12, no. 11: 3336. 10.3390/cancers12113336. PMC7698080 33187272

[fsn372329-bib-0015] Bhansali, S. , S. Khatri , and V. Dhawan . 2019. “ *Terminalia arjuna* Bark Extract Impedes Foam Cell Formation.” Lipids in Health and Disease 18: 195. 10.1186/s12944-019-1119-z. PMC6842518 31706299

[fsn372329-bib-0017] Bharani, A. , A. Ganguli , and K. D. Bhargava . 2002. “Efficacy of *Terminalia arjuna* in Chronic Stable Angina: A Double‐Blind, Crossover Study.” Indian Heart Journal 54, no. 2: 170–175. 12086380

[fsn372329-bib-0018] Bhatia, J. 2014. “Arjunolic Acid: A Potent Cardioprotective Triterpenoid From *Terminalia arjuna* .” Phytomedicine 21, no. 3: 359–366.

[fsn372329-bib-0019] Bhujel, M. , S. Kaloniya , D. Jain , L. Sripada , A. Bajaj , and G. N. Rao . 2025. “Amide Derivative of Arjunolic Acid TA‐1, 4‐BiP Enhances ROS‐Mediated Apoptosis in Colorectal Cancer Cells.” Current Medicinal Chemistry 32: 9756–9774. 10.2174/0109298673348718250109142630. 39871569

[fsn372329-bib-0020] Blagov, A. V. , V. A. Orekhova , V. N. Sukhorukov , A. A. Melnichenko , and A. N. Orekhov . 2023. “Potential Use of Antioxidant Compounds for the Treatment of Inflammatory Bowel Disease.” Pharmaceuticals 16, no. 8: 1150. 10.3390/ph16081150. PMC10458684 37631065

[fsn372329-bib-0022] Chaemsupaphan, T. , A. Arzivian , and R. W. Leong . 2025. “Comprehensive Care of Ulcerative Colitis: New Treatment Strategies.” 10.1080/17474124.2025.2457451. 39865726

[fsn372329-bib-0023] Chatha, S. A. , A. I. Hussain , R. Asad , M. Majeed , and N. Aslam . 2014. “Bioactive Components and Antioxidant Properties of *Terminalia arjuna* L. Extracts.” Journal of Food Processing & Technology 5, no. 298: 2. 10.4172/2157-7110.1000298.

[fsn372329-bib-0024] Chaudhari, G. M. , and R. T. Mahajan . 2015. “Comprehensive Study on Pharmacognostic, Physico and Phytochemical Evaluation of *Terminalia arjuna* Roxb. Stem Bark.” Journal of Pharmacognosy and Phytochemistry 4, no. 3: 186.

[fsn372329-bib-0026] Chaudhary, R. , and S. Sharma . 2020. “ *Terminalia arjuna*: A Promising Cardiotonic Drug.” International Journal of Pharmaceutical Sciences and Research 11, no. 7: 3013–3021.

[fsn372329-bib-0028] Cota, D. , S. Mishra , and S. Shengule . 2019. “Beneficial Role of *Terminalia arjuna* Hydro‐Alcoholic Extract in Colitis and Its Possible Mechanism.” Journal of Ethnopharmacology 230: 117–125. 10.1016/j.jep.2018.10.020. 30367989

[fsn372329-bib-0029] Cota, D. , S. Mishra , and S. Shengule . 2020. “Arjunarishta Alleviates Experimental Colitis via Suppressing Proinflammatory Cytokine Expression, Modulating Gut Microbiota and Enhancing Antioxidant Effect.” Molecular Biology Reports 47: 7049–7059. 10.1007/s11033-020-05766-z. 32885365

[fsn372329-bib-0030] Cota, D. L. , S. Mishra , S. A. Shengule , and D. Patil . 2022. “Assessment of In Vitro Biological Activities of *Terminalia arjuna* Roxb. Bark Extract and Arjunarishta in Inflammatory Bowel Disease and Colorectal Cancer.” Indian Journal of Experimental Biology 58, no. 5: 306–313.

[fsn372329-bib-0031] Dandu, S. S. P. , G. Sravanthi , S. Mohammed , S. Narahari , S. L. Sistla , and R. R. N. Reddy . 2020. “ *Terminalia arjuna*—A Possible Alternative to Commercial Mouthwashes Against Periodontopathic Bacteria: An In Vitro Study.” Journal of Dr NTR University of Health Sciences 9, no. 2: 98–102. 10.4103/JDRNTRUHS.JDRNTRUHS_81_20.

[fsn372329-bib-0032] Das, K. , S. Roy , S. Mandal , et al. 2016. “Renal Protection by Isolated Phytocompounds From *Terminalia arjuna* Bark Fraction on Dehydration Induced Uremia, Oxidative Stress and Kidney Disease Rats.” International Journal of Recent Scientific Research 7, no. 10: 13,787–13,795.

[fsn372329-bib-0033] Das, K. , L. Samanta , and G. B. N. Chainy . 2010. “Protective Effect of Aqueous Extract of *Terminalia arjuna* Against Dehydration‐Induced Oxidative Stress in Rats.” Journal of Young Pharmacists 2, no. 3: 194–198.

[fsn372329-bib-0034] Debnath, S. , D. Dey , S. Hazra , S. Ghosh , R. Ray , and B. Hazra . 2013. “Antibacterial and Antifungal Activity of *Terminalia arjuna* Wight & Arn. Bark Against Multi‐Drug Resistant Clinical Isolates.” Journal of Coastal Life Medicine 1, no. 4: 315–321. 10.12980/JCLM.1.2013C757.

[fsn372329-bib-0035] Devi, K. , J. Paulraj , R. S. George , R. Shanmugam , S. Maiti , and K. Devi . 2024. “A Comparative In Vitro Analysis of Antimicrobial Effectiveness and Compressive Resilience in Chirata and *Terminalia arjuna* Modified Glass Ionomer Cement.” Cureus 16, no. 1: e52198. 10.7759/cureus.52198. PMC10859781 38347981

[fsn372329-bib-0036] Devi, R. S. , M. Kist , G. Vani , and C. S. S. Devi . 2008. “Effect of Methanolic Extract of *Terminalia arjuna* Against *Helicobacter pylori* 26,695 Lipopolysaccharide‐Induced Gastric Ulcer in Rats.” Journal of Pharmacy and Pharmacology 60, no. 4: 505–514. 10.1211/jpp.60.4.0014. 18380924

[fsn372329-bib-0037] Devi, R. S. , S. Narayan , G. Vani , and C. S. S. Devi . 2007. “Gastroprotective Effect of *Terminalia arjuna* Bark on Diclofenac Sodium Induced Gastric Ulcer.” Chemico‐Biological Interactions 167, no. 1: 71–83. 10.1016/j.cbi.2007.01.011. 17327128

[fsn372329-bib-0038] Dube, N. , C. Nimgulkar , and D. K. Bharatraj . 2017. “Validation of Therapeutic Anti‐Inflammatory Potential of *Terminalia arjuna* in Animal Models.” Journal of Traditional and Complementary Medicine 7, no. 4: 452–457. 10.1016/j.jtcme.2016.11.006. PMC5634724 29034188

[fsn372329-bib-0039] Dutta, A. , and M. Das . 2023. “ *Terminalia arjuna* and Cardiovascular Protection: A Comprehensive Overview.” In Ancient and Traditional Foods, Plants, Herbs and Spices Used in Cardiovascular Health and Disease, 93–110. CRC Press.

[fsn372329-bib-0040] Dwivedi, S. 2007. “ *Terminalia arjuna* Wight & Arn.: A Useful Drug for Cardiovascular Disorders.” Journal of Ethnopharmacology 114, no. 2: 114–129. 10.1016/j.jep.2007.08.003. 17875376

[fsn372329-bib-0180] Dwivedi, S. , and D. Chopra . 2014. “Revisiting *Terminalia arjuna*—An Ancient Cardiovascular Drug.” Journal of Traditional and Complementary Medicine 4, no. 4: 224–231. 10.4103/2225-4110.139103. PMC4220499 25379463

[fsn372329-bib-0043] Elsherbiny, N. M. , M. A. Eladl , and M. M. Al‐Gayyar . 2016. “Renal Protective Effects of Arjunolic Acid in Cisplatin‐Induced Nephrotoxicity.” General Pharmacology 103: 109–118. 10.1016/j.cyto.2015.10.010. 26517155

[fsn372329-bib-0044] Elwekeel, A. , E. Amin , A. Moawad , and M. H. Hassan . 2024. “Comparative GC/MS Analysis, Antioxidant and Anticholinesterase Study for the Alcoholic Extracts of Leaves, Flowers and Bark of *Terminalia arjuna* (Roxb).” Pharmaceutical Chemistry Journal 58, no. 1: 112–118. 10.1007/s11094-024-03124-y.

[fsn372329-bib-0045] Engle, T. 2025. “Formulation and Evaluation of Gutika Using *Terminalia arjuna* Extract for Antidiabetic Activity.” International Journal of Pharmaceutical Sciences 3, no. 4: 1222–1234. 10.5281/zenodo.15188402.

[fsn372329-bib-0046] Garg, A. N. , P. T. Gajbhiye , and R. P. Choudhury . 2017. “INAA of Essential Micronutrients in *Terminalia arjuna* Bark Powder: A Versatile Heart Tonic.” Journal of Radioanalytical and Nuclear Chemistry 314, no. 3: 1539–1545. 10.1007/s10967-017-5564-8.

[fsn372329-bib-0047] Ghosh, J. , and P. C. Sil . 2013. “Arjunolic Acid: A New Multifunctional Therapeutic Promise of Alternative Medicine.” Biochimie 95, no. 6: 1098–1109. 10.1016/j.biochi.2013.01.016. 23402784

[fsn372329-bib-0048] Goel, S. , and K. Tarvinderjeet . 2013. “Nutritional Composition of Medicinal Plants Commonly Grown in the Kurukshetra District, Haryana, India.” Malaysian Journal of Nutrition 19, no. 2: 261–270.

[fsn372329-bib-0049] Gorde, P. , and A. Ganjale . 2020. “Development of Nutra Tea Using *Ficus racemosa* and *Terminalia arjuna* Bark Blends With Black Tea.” International Journal of Food Science and Nutrition 5, no. 6: 17–20.

[fsn372329-bib-0050] Gouthamchandra, K. , H. V. Sudeep , A. Sathish , L. H. Basavegowda , T. P. Prasanna Kumar , and S. P. Kodimule . 2024. “Cardioprotective Potential of Cardiboost, a Standardized Extract From *Terminalia arjuna* Bark Against H_2_O_2_ Induced Oxidative Stress in H9c2 Cardiomyocytes.” Journal of Young Pharmacists 16, no. 1: 10–16. 10.5530/jyp.2024.16.2.

[fsn372329-bib-0051] Greco, G. , E. Turrini , M. Tacchini , I. Maresca , and C. Fimognari . 2021. “The Alcoholic Bark Extract of *Terminalia arjuna* Exhibits Cytotoxic and Cytostatic Activity on Jurkat Leukemia Cells.” Venoms and Toxins 1, no. 1: 56–66. 10.2174/2666121701999200601170928.

[fsn372329-bib-0052] Grewal, J. , V. Kumar , Y. Gandhi , et al. 2023. “Current Perspective and Mechanistic Insights on Bioactive Plant Secondary Metabolites for the Prevention and Treatment of Cardiovascular Diseases.” Cardiovascular & Haematological Disorders‐Drug Targetsrug Targets‐Cardiovascular & Hematological Disorders 23, no. 3: 157–176. 10.2174/011871529X262371231009132426. 37921163

[fsn372329-bib-0054] Gruber, S. F. , M. A. Gallagher , M. S. Malik , and M. A. K. Hall . 2024. “More Than Just Stool: Hypereosinophilic Syndrome Presenting as Persistent Diarrhea in a Patient With Ulcerative Colitis.” Journal of Brown Hospital Medicine 3, no. 4: 9–12. 10.56305/001c.122954. PMC11864473 40026549

[fsn372329-bib-0056] Hafiz, F. B. , N. M. Towfique , M. K. Sen , S. N. Sima , B. S. Azhar , and M. M. Rahman . 2014. “A Comprehensive Ethno‐Pharmacological and Phytochemical Update Review on Medicinal Plant of *Terminalia arjuna* Roxb. of 4angladesh.” Scholars Academic Journal of Pharmacy 3: 19–25.

[fsn372329-bib-0057] Haque, R. , S. Paul , M. T. Sultan , et al. 2025. “Supplementation of Arjun (*Terminalia arjuna*) Bark Powder Prevented Oxidative Stress and Enhanced Antioxidants in Kidneys on Isoproterenol‐Treated Swiss Albino Mice Model.” Clinical Nutrition Open Science 60: 66–77. 10.1016/j.nutos.2025.01.013.

[fsn372329-bib-0058] Harshitha, K. , and U. S. Gorla . 2021. “Protective Effect of *Terminalia arjuna* Against DBTC Induced Pancreatic Cancer in Male Wistar Rats.” Journal of Pharmaceutical Research International 33, no. 46A: 248–256. 10.9734/JPRI/2021/v33i46A32863.

[fsn372329-bib-0059] Hasan, M. M. , P. Madhavan , N. A. Ahmad Noruddin , et al. 2023. “Cardioprotective Effects of Arjunolic Acid in LPS‐Stimulated H9C2 and C2C12 Myotubes via the MyD88‐Dependent TLR4 Signaling Pathway.” Pharmaceutical Biology 61, no. 1: 1135–1151. 10.1080/13880209.2023.2230251. PMC10375937 37497554

[fsn372329-bib-0060] He, Q. , W. Wei , J. Li , Z. Chen , and H. Zhang . 2025. “Ulcerative Colitis‐Associated Diffuse Enteritis Without Prior Colectomy: A Case Report.” Medicine 104, no. 25: e42924. 10.1097/MD.0000000000042924. PMC12187356 40550068

[fsn372329-bib-0061] Huddar, S. , N. S. Ullegaddi , and K. Vaishalee . 2024. “Research Article A Pharmacological Study of *Arjuna termanalia* (arjuna)–A Brief Study.”

[fsn372329-bib-0062] Imamovna, K. K. 2025. “Modern Theories in the Etiology of Ulcerative Colitis: A Literature Review.” Modern American Journal of Medical and Health Sciences 1, no. 2: 39–49.

[fsn372329-bib-0063] Iqram, T. , D. Begum , F. Hossain , et al. 2025a. “Effect of *Terminalia arjuna* (arjun) Bark on Serum Aspartate Aminotransferase and Alanine Aminotransferase in Isoniazid Induced Hepatotoxicity in Wistar Albino Male Rats.” Eastern Medical College Journal 10, no. 1: 18–22. 10.3329/emcj.v10i1.82520.

[fsn372329-bib-0064] Iqram, T. , D. Begum , F. Hossain , et al. 2025b. “Effect of *Terminalia arjuna* on Isoniazid‐Induced Liver Damage in Rats.” Eastern Medical College Journal 10, no. 1: 18–22. 10.3329/emcj.v10i1.82520.

[fsn372329-bib-0065] Irfan, M. , M. S. A. Raheem , and R. K. Sharma . 2025. “Role of Unani Medicine in Geriatric Care: A Holistic Approach to Healthy Ageing.” International Journal of Medical Sciences and Pharma Research 11, no. 2: 32–41. 10.22270/ijmspr.v11i2.152.

[fsn372329-bib-0066] Jagtap, D. , J. Ambhore , S. Dapse , and V. Patel . 2024. “To Study the Antimicrobial and Antioxidant Potential of *Terminalia arjuna* Leaves and Bark Extracts.” International Journal of Pharmacy and Pharmaceutical Sciences 6, no. 2: 110–115. 10.33545/26647222.2024.v6.i2b.131.

[fsn372329-bib-0067] Jain, N. K. , and N. Singh . 2023. “Hepatoprotective Role of Herbs and Herbal.” In Plant‐Derived Hepatoprotective Drugs, 81. BENTHAM SCIENCE PUBLISHERS.

[fsn372329-bib-0069] Jaiswal, K. , T. Thakur , N. Mishra , and A. Kumar . 2021. “Pharmacological Approach of *Terminalia arjuna*: A Review.” Plant Cell Biotechnology and Molecular Biology 22, no. 57–58: 1–15. https://ikprress.org/index.php/PCBMB/article/view/7065.

[fsn372329-bib-0071] Jude, S. , A. C. K. Varma , S. Kuttappan , and A. Amalraj . 2022. “Ayurvedic and Traditional Systems of Medicine in Clinical Trials: An Overview.” In Chemistry, Biological Activities and Therapeutic Applications of Medicinal Plants in Ayurveda. Royal Society of Chemistry. 10.1039/9781839166211-00391.

[fsn372329-bib-0072] Kalola, J. , and M. Rajani . 2006. “Extraction and TLC Desitometric Determination of Triterpenoid Acids (Arjungenin, Arjunolic Acid) From *Terminalia arjuna* Stem Bark Without Interference of Tannins.” Chromatographia 63, no. 9: 475–481. 10.1365/s10337-006-0772-3.

[fsn372329-bib-0073] Kannappan, P. , R. Senthil Ganesh , S. Sivanesan , R. Vijayaraghavan , and M. Swaminathan . 2020. “A Study on the Inhibition of Oxidative Stress, Inflammation and Apoptosis by *Terminalia arjuna* Against Acetaminophen‐Induced Hepatotoxicity in Wistar Albino Rats.” Indian Journal of Biochemistry and Biophysics 57, no. 1: 51–57.

[fsn372329-bib-0074] Kanthe, P. S. , B. S. Patil , and K. K. Das . 2022. “ *Terminalia arjuna* Supplementation Ameliorates High Fat Diet‐Induced Oxidative Stress in Nephrotoxic Rats.” Journal of Basic and Clinical Physiology and Pharmacology 33, no. 4: 409–417. 10.1515/jbcpp-2020-0106. 33743558

[fsn372329-bib-0075] Kaplan, G. G. 2025. “The Global Burden of Inflammatory Bowel Disease: From 2025 to 2045.” Nature Reviews Gastroenterology & Hepatology 22, no. 10: 708–720. 10.1038/s41575-025-01097-1. 40681759

[fsn372329-bib-0076] Kapoor, D. , R. Vijayvergiya , and V. Dhawan . 2014. “ *Terminalia arjuna* in Coronary Artery Disease: Ethnopharmacology, Pre‐Clinical, Clinical & Safety Evaluation.” Journal of Ethnopharmacology 155, no. 2: 1029–1045. 10.1016/j.jep.2014.06.056. 25014508

[fsn372329-bib-0078] Khaliq, F. , and M. Fahim . 2018. “Role of *Terminalia arjuna* in Improving Cardiovascular Functions: A Review.” Indian Journal of Physiology and Pharmacology 62, no. 1: 8–19.

[fsn372329-bib-0079] Khan, I. , Z. Ullah , A. A. Shad , M. Fahim , and M. Öztürk . 2022. “In Vitro Antioxidant, Anticholinesterase Inhibitory, and Antimicrobial Activity Studies of *Terminalia chebula* (Retz) and *Terminalia arjuna* (Roxb).” South African Journal of Botany 146: 395–400. 10.1016/j.sajb.2021.11.016.

[fsn372329-bib-0080] Kumar, R. , S. Singh , and A. K. Meena . 2016. “Quality Assessment of *Terminalia arjuna* (Roxb. Ex DC.) Stem Bark Used as Cardio‐Protective Drug and Its Scientific Identification by HPTLC Using Arjunic Acid as Marker.” International Journal of Pharmacognosy and Phytochemical Research 8, no. 4: 597–603.

[fsn372329-bib-0081] Kumar, V. , N. Sharma , R. Saini , et al. 2023. “Therapeutic Potential and Industrial Applications of *Terminalia arjuna* Bark.” Journal of Ethnopharmacology 310: 116352. 10.1016/j.jep.2023.116352. 36933876

[fsn372329-bib-0174] Li, J. , B. Zou , Y. H. Yeo , et al. 2019. “Prevalence, Incidence, and Outcome of Non‐Alcoholic Fatty Liver Disease in Asia, 1999–2019: A Systematic Review and Meta‐Analysis.” Lancet Gastroenterology & Hepatology 4, no. 5: 389–398. 10.1016/S2468-1253(19)30039-1 30902670

[fsn372329-bib-0175] Liu, J. , Y. Yang , J. Ding , B. Zhu , and W. Gao . 2019. “Microfibers: A Preliminary Discussion on Their Definition and Sources.” Environmental Science and Pollution Research 26, no. 28: 29497–29501. 10.1007/s11356-019-06265-w. 31444725

[fsn372329-bib-0082] Liu, X. F. , J. H. Shao , Y. T. Liao , et al. 2023. “Regulation of Short‐Chain Fatty Acids in the Immune System.” Frontiers in Immunology 14: 1186892. 10.3389/fimmu.2023.1186892. PMC10196242 37215145

[fsn372329-bib-0083] Luna, R. K. , N. S. Thakur , and V. Kumar . 2011. “Growth Performance of Twelve New Clones of Poplar in Punjab, India.” Indian Journal Of Ecology 38: 107–109.

[fsn372329-bib-0084] Maaz, M. , M. T. Sultan , K. Ahmad , et al. 2025. “ *Citrullus colocynthis* And Human Health: Transforming Nature Bitterness to Healing Medicine.” Applied Food Research 101: 477.

[fsn372329-bib-0086] Mahajan, B. 2024. “Therapeutic Potential of Indian Medicinal Plants in Diabetic Nephropathy.” Insight 1, no. 3: 30–38. 10.5281/zenodo.15421214.

[fsn372329-bib-0088] Maulik, S. K. , V. Wilson , S. Seth , et al. 2016. “Clinical Efficacy of Water Extract of Stem Bark of *Terminalia arjuna* (Roxb. Ex DC.) Wight & Arn. In Patients of Chronic Heart Failure: A Double‐Blind, Randomized Controlled Trial.” Phytomedicine 23, no. 11: 1211–1219. 10.1016/j.phymed.2016.02.007. 26988798

[fsn372329-bib-0089] Meena, D. K. , B. K. Das , A. K. Sahoo , N. P. Sahu , P. P. Srivastava , and S. Borah . 2024. “ *Terminalia arjuna* Bark Powder as a Potential Immunomodulator in *Labeo rohita* : Enhanced Hematological, Adaptive, and Humoral Responses Against Bacterial Pathogens and Concordant Liver Histomorphology.” Pathogens 13, no. 4: 295. 10.3390/pathogens13040295. PMC11054661 38668250

[fsn372329-bib-0090] Meena, D. K. , B. K. Das , A. K. Sahoo , and K. Satvik . 2025. “ *Terminalia arjuna* Bark Powder as a Nutritive Additive for Enhancing Fish Health and Environmental Sustainability.” Discover Food 5, no. 1: 1–13. 10.1007/s44187-025-00329-2.

[fsn372329-bib-0091] Meena, D. K. , A. K. Sahoo , H. Chowdhury , et al. 2020. “Effects of Extraction Methods and Solvent Systems on Extract Yield, Proximate Composition and Mineral Profiling of *Terminalia arjuna* Dry Powders and Solvent Extracts.” Journal of Innovations in Pharmaceutical and Biological Sciences 7, no. 1: 22–31.

[fsn372329-bib-0092] Meena, D. K. , A. K. Sahoo , P. P. Srivastava , et al. 2021. “On Valorization of Solvent Extracts of *Terminalia arjuna* (arjuna) Upon DNA Scission and Free Radical Scavenging Improves Coupling Responses and Cognitive Functions Under In Vitro Conditions.” Scientific Reports 11, no. 1: 10,656. 10.1038/s41598-021-88710-w. PMC8137696 34017022

[fsn372329-bib-0182] Mishra, S. , P. K. Sahu , V. Agarwal , and N. Singh . 2021. “Exploiting Endophytic Microbes as Micro‐Factories for Plant Secondary Metabolite Production.” Applied Microbiology and Biotechnology 105, no. 18: 6579–6596. 10.1007/s00253-021-11527-0. 34463800

[fsn372329-bib-0172] Mishra, S. K. , T. Sanyal , P. Kundu , et al. 2025. “Microplastics as Emerging Carcinogens: From Environmental Pollutants to Oncogenic Drivers.” Molecular Cancer 24, no. 1: 248. 10.1186/s12943-025-02409-4 PMC12505851 41063167

[fsn372329-bib-0095] Mittal, A. , S. Tandon , S. K. Singla , and C. Tandon . 2016. “Mechanistic Insights Into the Antilithiatic Proteins From *Terminalia arjuna*: A Proteomic Approach in Urolithiasis.” PLoS One 11, no. 9: e0162600. 10.1371/journal.pone.0162600. PMC5029924 27649531

[fsn372329-bib-0096] Mohanty, I. R. , M. Borde , C. S. Kumar , and U. Maheshwari . 2019. “DPP‐IV Inhibitory Activity of *Terminalia arjuna*—Clinical/Experimental Findings.” Journal of Ethnopharmacology 228: 143–149. 10.1016/j.phymed.2018.09.195.

[fsn372329-bib-0097] Mohanty, I. R. , M. Borde , and U. Maheshwari . 2019. “Dipeptidyl Peptidase IV Inhibitory Activity of *Terminalia arjuna* Attributes to Its Cardioprotective Effects in Experimental Diabetes: In Silico, In Vitro and In Vivo Analyses.” Phytomedicine 57: 158–165. 10.1016/j.phymed.2018.09.195. 30668318

[fsn372329-bib-0098] Mukherjee, D. 2020. “ *Terminalia arjuna* Protects Against Methotrexate‐Induced Intestinal Mucositis Through NF‐κB Pathway Inhibition.” Pharmaceutical Biology 58, no. 1: 890–898.

[fsn372329-bib-0099] Muro, P. , L. Zhang , S. Li , et al. 2024. “The Emerging Role of Oxidative Stress in Inflammatory Bowel Disease.” Frontiers in Endocrinology 15: 1390351. 10.3389/fendo.2024.1390351. PMC11284038 39076514

[fsn372329-bib-0100] Mutheeswaran, S. , P. Ganesan , and M. Muthukanagavel . 2025. “Nature's Cure: Innovative Herbal Approaches to Inflammatory Disease Management.” Journal of Anatomy and Physiology 1, no. 1: 1–5.

[fsn372329-bib-0102] Neurath, M. F. 2014. “Cytokines in Inflammatory Bowel Disease.” Nature Reviews. Immunology 14, no. 5: 329–342. 10.1038/nri3661. 24751956

[fsn372329-bib-0103] Ni, J. , G. D. Wu , L. Albenberg , and V. T. Tomov . 2017. “Gut Microbiota and IBD: Causation or Correlation?” Nature Reviews. Gastroenterology & Hepatology 14, no. 10: 573–584. 10.1038/nrgastro.2017.88. PMC5880536 28743984

[fsn372329-bib-0104] Noman, A. M. , M. T. Sultan , M. Maaz , et al. 2025. “Nutraceutical Potential of Anthocyanins: A Comprehensive Treatise.” Food Science & Nutrition 13, no. 5: e70164. 10.1002/fsn3.70164. PMC12050221 40330208

[fsn372329-bib-0105] Nunes, P. H. M. , M. d. C. C. Martins , R. d. C. M. Oliveira , et al. 2014. “Gastric Anti‐Ulcerogenic and Hypokinetic Activities of *T. arjuna* Bark Methanol Extract.” International Journal of Molecular Sciences 15, no. 10: 18,164–18,176. 10.1155/2014/261745.

[fsn372329-bib-0181] Olchowik‐Grabarek, E. , S. Sękowski , A. Kwiatek , et al. 2022. “The Structural Changes in the Membranes of *Staphylococcus aureus* Caused by Hydrolysable Tannins Witness Their Antibacterial Activity.” Membranes 12, no. 11: 1124. 10.3390/membranes12111124. PMC9698758 36363679

[fsn372329-bib-0106] Padmavathy, B. , B. S. Ebinezer , S. Amalraj , et al. 2024. “Cytotoxicity Effects of *Terminalia arjuna* Bark‐Derived Nano‐ZnO on MCF 7 Cells and Enhanced Anti‐Breast Cancer Potency of Its Phytochemicals Upon Zn‐Capping.” Materials Chemistry and Physics 328: 130030. 10.1016/j.matchemphys.2024.130030.

[fsn372329-bib-0107] Palanivelu, P. , V. Jagadesan , R. Vijayaraghavan , D. Rajkumar , M. Krishnamoorthy , and S. Rajakumar . 2023. “ *Terminalia arjuna* Bark Extract Reduces High‐Fat Diet Induced Cardiac Damage in Wistar Rats by Altering Biochemical and Histological Parameters.” Indian Journal of Pharmaceutical Education and Research 57, no. 1: 83–93. 10.5530/001954641032.

[fsn372329-bib-0108] Papakyriakopoulou, P. , N. Velidakis , E. Khattab , G. Valsami , I. Korakianitis , and N. P. Kadoglou . 2022. “Potential Pharmaceutical Applications of Quercetin in Cardiovascular Diseases.” Pharmaceuticals 15, no. 8: 1019. 10.3390/ph15081019. PMC9412669 36015169

[fsn372329-bib-0109] Parmar, H. S. , S. Panda , R. Jatwa , and A. Kar . 2006. “Cardio‐Protective Role of *T. arjuna* Possibly Mediated via Thyroid Hormones.” Pharmazie 61, no. 9: 793–795. 17020158

[fsn372329-bib-0110] Parveen, K. , P. Khan , and W. A Siddiqui . 2011. “Antidiabetic effects afforded by Terminalia arjuna in high fat‐fed and streptozotocin‐induced type 2 diabetic rats.” International Journal of Diabetes and Metabolism 19, no. 1: 23–33. 10.1159/000497707.

[fsn372329-bib-0111] Patel, V. R. , A. K. Gupta , J. Dwivedi , et al. 2025. “Anthocephalus Indicus and *Terminalia arjuna* Combination Ameliorates Hematological and Histopathological Changes in a Rat Model of Metabolic Syndrome.” Cureus 17, no. 9: e93523. 10.7759/cureus.93523. PMC12572365 41179088

[fsn372329-bib-0112] Patil, U. H. , and D. K. Gaikwad . 2011. “Pharmacognostical Evaluation of Stem Bark of *Terminalia arjuna* .” International Journal of Pharmacy and Pharmaceutical Sciences 3, no. Suppl. 4: 98–102.

[fsn372329-bib-0113] Pineton de Chambrun, G. , L. Peyrin‐Biroulet , M. Lémann , and J. F. Colombel . 2010. “Clinical implications of mucosal healing for the management of IBD.” Nature reviews Gastroenterology & hepatology 7, no. 1: 15–29. 10.1038/nrgastro.2009.203. 19949430

[fsn372329-bib-0115] Prakash, V. , N. Goel , K. R. Giri , and A. Goel . 2024. “Clinical Study Evaluating Antihyperglycemic Efficacy and Safety of *Terminalia arjuna* Versus Sitagliptin in Type 2 Diabetes Mellitus Patients.” Journal of Endocrine Research 12, no. 1: 23–31. 10.6026/9732063002001862. PMC11993424 40230891

[fsn372329-bib-0117] Priya, N. , K. C. Mathur , A. Sharma , R. P. Agrawal , V. Agarwal , and J. Acharya . 2019. “Effect of *T. arjuna* on Platelet Count & Lipid Profile in CAD Patients.” Advances in Human Biology 9, no. 1: 98–101. 10.4103/AIHB.AIHB_8_18.

[fsn372329-bib-0118] Quesada‐Granados, J. J. , J. Plaza‐Díaz , A. Molina‐López , and M. S. Abellán‐Ruiz . 2023. “Comparative Analysis of Traditional Oriental Herbal Fruits as Potential Sources of Polyphenols and Minerals for Nutritional Supplements.” Molecules 28, no. 6: 2682. 10.3390/molecules28062682. PMC10058731 36985653

[fsn372329-bib-0119] Rahman, M. A. , and R. Amin . 2003. Monograph on Arjun (Terminalia arjuna). https://www.academia.edu/43438854/Monograph_on_Arjun_Terminalia_arjuna.

[fsn372329-bib-0120] Rahmat, S. , A. B. Mannan , M. K. Prottasha , et al. 2024. “Hepatoprotective and Anti‐Hyperlipidemic Effects of Ethanolic Extract of *Terminalia arjuna* in a High‐Fat‐Induced Hyperlipidemic Rat Model.” Asian Journal of Advanced Research and Reports 18, no. 9: 200–209. 10.9734/ajarr/2024/v18i9744.

[fsn372329-bib-0177] Rai, N. K. , A. Ashok , and B. R. Akondi . 2020. “Consequences of Chemical Impact of Disinfectants: Safe Preventive Measures Against COVID‐19.” Critical Reviews in Toxicology 50, no. 6: 513–520. 10.1080/10408444.2020.1790499 32729370

[fsn372329-bib-0178] Rai, P. K. , C. Sonne , and K. H. Kim . 2023. “Heavy Metals and Arsenic Stress in Food Crops: Elucidating Antioxidative Defense Mechanisms in Hyperaccumulators for Food Security, Agricultural Sustainability, and Human Health.” Science of the Total Environment 874: 162327. 10.1016/j.scitotenv.2023.162327. 36813200

[fsn372329-bib-0121] Raj, C. D. , M. M. Shabi , J. Jipnomon , et al. 2012. “ *Terminalia arjuna*'s Antioxidant Effect in Isolated Perfused Kidney.” Research in Pharmaceutical Sciences 7, no. 3: 181–188. PMC3501927 23181096

[fsn372329-bib-0122] Rajaram, V. , A. Namasivayam , R. Shanmugam , et al. 2024. “ *Terminalia arjuna*: An Overview of Its Magical Properties.” Bioinformation 20, no. 12: 2080–2085. 10.6026/9732063002002080. PMC11993416 40230946

[fsn372329-bib-0123] Ramesh, A. S. , J. G. Christopher , R. Radhika , C. R. Setty , and V. Thankamani . 2012. “Isolation, Characterisation and Cytotoxicity Study of Arjunolic Acid From *Terminalia arjuna* .” Natural Product Research 26, no. 16: 1549–1552. 10.1080/14786419.2011.566870. 21732908

[fsn372329-bib-0124] Ramesh, P. , and A. Palaniappan . 2023. “ *Terminalia arjuna*, a Cardioprotective Herbal Medicine–Relevancy in the Modern Era of Pharmaceuticals and Green Nanomedicine—A Review.” Pharmaceuticals 16, no. 1: 126. 10.3390/ph16010126. PMC9865560 36678623

[fsn372329-bib-0125] Rana, M. S. , R. Walia , A. Dixit , and K. Raina . 2016. “To Compare and Evaluate the Anti‐Inflammatory Efficacy of *Terminalia arjuna* (Aqueous Extract of Bark) With Diclofenac Sodium on Rats.” International Journal of Basic & Clinical Pharmacology 5, no. 3: 692–695. 10.18203/2319-2003.ijbcp20161502.

[fsn372329-bib-0126] Rana, S. V. , S. Sharma , K. K. Prasad , S. K. Sinha , and K. Singh . 2014. “Role of Oxidative Stress & Antioxidant Defence in Ulcerative Colitis Patients From North India.” Indian Journal of Medical Research 139, no. 4: 568–571. PMC4078495 24927343

[fsn372329-bib-0127] Raza, H. , M. T. Sultan , K. Ahmad , et al. 2025. “ *Hibiscus rosa‐sinensis* : A Multifunctional Flower Bridging Nutrition, Medicine, and Molecular Therapeutics.” Food Science & Nutrition 13, no. 12: e71254. 10.1002/fsn3.71254. PMC12658387 41323831

[fsn372329-bib-0128] Roodagi, M. 2015. “Studies on Soil Organic Carbon Sequestration Under Selected Tree Plantations And Agroforestry Systems Of Northern Transition And Hill Zones Of Karnataka.”

[fsn372329-bib-0129] Rybakovsky, E. , M. C. Valenzano , R. Deis , K. M. DiGuilio , S. Thomas , and J. M. Mullin . 2017. “Improvement of Human‐Oral‐Epithelial‐Barrier Function and of Tight Junctions by Micronutrients.” Journal of Agricultural and Food Chemistry 65, no. 50: 10950–10958. 10.1021/acs.jafc.7b04203 29172516

[fsn372329-bib-0130] Saha, A. , V. M. Pawar , and S. Jayaraman . 2012. “Characterisation of Polyphenols in *Terminalia arjuna* Bark Extract.” Indian Journal of Pharmaceutical Sciences 74, no. 4: 339–347. 10.4103/0250-474X.107067. PMC3630729 23626389

[fsn372329-bib-0131] Sahoo, A. R. 2022. “Total Phenolic and Flavonoid Content in Different Parts of *Terminalia arjuna* (Roxb.) Wight and Arn and Its Variation With Storage of Bark.” Doctoral Dissertation, Department of Forest Products and Utilisation, OUAT, Bhubaneswar.

[fsn372329-bib-0134] Sarma, R. M. , B. Subramanian , P. Tamilmaran , and G. Ramakrishnan . 2019. “Anti‐Inflammatory and Anti‐Granuloma Effect of the Extract of the Leaf of *Terminalia arjuna* (Roxb.) Wight & Arn.” Journal of Pharmaceutical Sciences and Research 11, no. 11: 3579–3586.

[fsn372329-bib-0135] Saxena, M. , U. Faridi , R. Mishra , et al. 2007. “Cytotoxic Agents From *Terminalia arjuna* .” Planta Medica 73, no. 14: 1486–1490. 10.1055/s-2007-990258. 18008199

[fsn372329-bib-0136] Shankar, K. M. P. 2024. “Effectiveness of Herbal *T. arjuna* in Chronic Venous Insufficiency.” Phlebology 39, no. 6: 463–470. 10.1016/j.jvn.2023.11.009.

[fsn372329-bib-0179] Sharma, A. , P. Jaiswal , Y. Kerakhan , et al. 2021. “Liver Disease and Outcomes Among COVID‐19 Hospitalized Patients—A Systematic Review and Meta‐Analysis.” Annals of Hepatology 21: 100273. 10.1016/j.aohep.2020.10.001. PMC7566821 33075578

[fsn372329-bib-0137] Sharma, R. 2022. “Arjunolic Acid Ameliorates TNBS‐Induced Ulcerative Colitis in Rats.” Inflammopharmacology 30, no. 2: 725–737.

[fsn372329-bib-0139] Sherif, I. O. 2021. “Hepatoprotective Effect of Arjunolic Acid Against Cisplatin‐Induced Hepatotoxicity: Targeting Oxidative Stress, Inflammation, and Apoptosis.” Basic Clinical Pharmacology & Toxicology 35: e22714. 10.1002/jbt.22714. 33491850

[fsn372329-bib-0140] Siddiqui, M. T. , R. Kasiraj , and M. Naseer . 2025. “Medical Management of Ulcerative Colitis and Crohn's Disease—Strategies for Inducing and Maintaining Remission.” Surgical Clinics 105, no. 2: 435–454. 10.1016/j.suc.2024.10.007. 40015826

[fsn372329-bib-0141] Singh, A. , V. Bajpai , S. Kumar , K. R. Sharma , and B. Kumar . 2016. “Profiling of Gallic and Ellagic Acid Derivatives in Different Plant Parts of *Terminalia arjuna* by HPLC‐ESI‐QTOF‐MS/MS.” Natural Product Communications 11, no. 2: 1934578X1601100227. 10.1177/1934578X1601100227. 27032211

[fsn372329-bib-0142] Singh, C. , V. P. Singh , A. K. Verma , P. Umaraw , and V. Vihan . 2025. “Exploring the Antioxidant and Antimicrobial Efficacy of Ethanolic Extract of *Terminalia arjuna* in Meat Batter.” Food Science and Technology International 32: 603–616. 10.1177/10820132251338165. 40350572

[fsn372329-bib-0143] Singh, S. , S. K. Verma , and S. Kumar . 2017. “Analysis of Anti‐Cancer Potential of *Terminalia arjuna* .” International Journal of Advanced Scientific Research and Management 2, no. 11: 82–87.

[fsn372329-bib-0144] Sivalokanathan, S. , M. Ilayaraja , and M. P. Balasubramanian . 2006. “Antioxidant Activity of *Terminalia arjuna* Bark Extract on N‐Nitrosodiethylamine Induced Hepatocellular Carcinoma in Rats.” Molecular and Cellular Biochemistry 281, no. 1–2: 87–93. 10.1007/s11010-006-0339-7. 16328960

[fsn372329-bib-0146] Stępnik, K. , W. Kukula‐Koch , and W. Płaziński . 2023. “Molecular and Pharmacokinetic Aspects of the Acetylcholinesterase‐Inhibitory Potential of the Oleanane‐Type Triterpenes and Their Glycosides.” Biomolecules 13, no. 9: 1357. 10.3390/biom13091357. PMC10526139 37759757

[fsn372329-bib-0147] Subramaniam, S. , R. Subramaniam , S. Rajapandian , S. Uthirapathy , and V. R. Gnanamanickam . 2011. “Anti‐Inflammatory and Anti‐Arthritic Efficacy of *Terminalia arjuna* Bark Extracts.” International Journal of Pharmacy and Pharmaceutical Sciences 3, no. 2: 188–192.

[fsn372329-bib-0148] Sultan, M. T. , M. S. Butt , R. Karim , et al. 2014. “Effect of *Nigella sativa* Fixed and Essential Oils on Antioxidant Status, Hepatic Enzymes, and Immunity in Streptozotocin Induced Diabetes Mellitus.” BMC Complementary and Alternative Medicine 14, no. 1: 193. 10.1186/1472-6882-14-193 PMC4077235 24939518

[fsn372329-bib-0149] Swain, R. K. , S. N. Tripathy , R. Rana , S. K. Patro , and S. Behera . 2023. “A Study on Phytochemical and Pharmacological Activity of *Terminalia arjuna*: A Review.” Research Journal of Pharmacognosy and Phytochemistry 15, no. 2: 111–117.

[fsn372329-bib-0150] Tahir, H. , M. N. Akhtar , A. K. Bishoyi , et al. 2025. “Nutritional Composition, Phytochemical Profile, Extraction Methods of Bioactive Components, and Health Benefits of *Terminalia arjuna* Bark.” eFood 6, no. 2: e70038. 10.1002/efd2.70038.

[fsn372329-bib-0151] Tahsin, M. R. , A. Sultana , M. S. M. Khan , et al. 2021. “An Evaluation of Pharmacological Healing Potentialities of *Terminalia arjuna* Against Several Ailments on Experimental Rat Models With an In Silico Approach.” Heliyon 7, no. 11: e08225. 10.1016/j.heliyon.2021.e08225. PMC8591345 34816025

[fsn372329-bib-0153] Timilsina, K. , A. Mishra , V. Patial , M. Gupta , V. Chaudhary , and D. Singh . 2024. “A Randomized Controlled Clinical Trial and Preclinical Efficacy of an Ayurvedic Formulation Arjuna Ksheerapaka Churna for Dyslipidemia: Arjuna Ksheerapaka Churna for Dyslipidemia.” Indian Journal of Traditional Knowledge 23, no. 6: 519–529.

[fsn372329-bib-0154] Tindemans, I. , M. E. Joosse , and J. N. Samsom . 2020. “Dissecting the Heterogeneity in T‐Cell Mediated Inflammation in IBD.” Cells 9, no. 1: 110. 10.3390/cells9010110. PMC7016883 31906479

[fsn372329-bib-0155] Toppo, E. , S. S. Darvin , S. Esakkimuthu , et al. 2018. “Curative Effect of Arjunolic Acid From *Terminalia arjuna* in Non‐Alcoholic Fatty Liver Disease Models.” Biomedicine & Pharmacotherapy 107: 979–988. 10.1016/j.biopha.2018.08.019. 30257410

[fsn372329-bib-0158] Tyagi, P. , and H. A. Khan . 2018. “Amelioration of oxidative stress in the joint tissue may be the basis for the antiarthritic activity of Terminalia arjuna bark extract.” International Journal of Rheumatic Diseases 21, no. 12: 2079–2088. 10.1111/1756-185X.12429. 25294686

[fsn372329-bib-0159] Ungaro, R. , S. Mehandru , P. B. Allen , L. Peyrin‐Biroulet , and J. F. Colombel . 2017. “Ulcerative colitis.” Lancet 389, no. 10080: 1756–1770. 10.1016/s0140-6736(16)32126-2. PMC6487890 27914657

[fsn372329-bib-0160] Upadhyay, J. , A. V. Sonaji , K. S. Trimbak , C. S. Ganesh , K. V. Pawar , and R. Kumar . 2024. “Role of Herbal Active Compound in Cardiac Failure Treatment.” Journal for Research in Applied Sciences and Biotechnology 3, no. 2: 83–102. 10.55544/jrasb.3.2.16.

[fsn372329-bib-0161] van der Post, S. , K. S. Jabbar , G. Birchenough , et al. 2019. “Structural Weakening of the Colonic Mucus Barrier Is an Early Event in Ulcerative Colitis Pathogenesis.” Gut 68, no. 12: 2142–2151. 10.1136/gutjnl-2018-317571 PMC6872445 30914450

[fsn372329-bib-0162] Vashistt, J. 2022. “Study on Natural Antimicrobial Compounds Against Bacterial Isolates.”

[fsn372329-bib-0163] Verma, A. , G. K. Vyas , and N. Nama . 2025. A Review on Anti‐Inflammatory Potential of *Terminalia arjuna* and its Application in Oral Suspension Formulation Cardiovascular Disorders.

[fsn372329-bib-0173] Verma, R. K. , N. Pal , S. Gupta , et al. 2025. “Novel Extraction Technique: Quantification of Major Phytoconstituents of Arjuna in Infused Edible Oil.” Natural Product Research 39, no. 2: 294–299. 10.1080/14786419.2023.2263899. 37799113

[fsn372329-bib-0165] Vijayalakshmi, R. , N. Ambalavanan , and S. Rajeshkumar . 2023. “Antimicrobial and Anti‐Inflammatory Activity of *Terminalia arjuna* .” Bioinformation 19, no. 2: 184. 10.6026/97320630019184. PMC10560303 37814683

[fsn372329-bib-0166] Wadhai, M. , D. Ayate , V. V. Ujjainkar , and A. U. Nimkar . 2025. “Studies on Arjun *Terminalia arjuna* (Roxb.) for Tannin and Ash Content in Vidarbha Region of Maharashtra, India.” Environment and Ecology 43, no. 2: 560–565. 10.60151/envec/TYLN5838.

[fsn372329-bib-0167] Wang, W. , Z. Ali , Y. H. Sheng , X. C. Li , and I. A. Khan . 2010. “Triterpenoids From the Bark of *Terminalia arjuna* (Combretaceae).” Planta Medica 76, no. 5: P45. 10.1055/s-0030-1251807. 20112178

[fsn372329-bib-0168] Zhang, Z. , M. Cao , Z. Shang , et al. 2025. “Research Progress on the Antibacterial Activity of Natural Flavonoids.” Antibiotics 14, no. 4: 334. 10.3390/antibiotics14040334. PMC12023951 40298463

[fsn372329-bib-0170] Zhang, Z. , L. Zuo , X. Song , et al. 2024. “Arjunolic Acid Protects the Intestinal Epithelial Barrier, Ameliorating Crohn's Disease‐Like Colitis by Restoring Gut Microbiota Composition and Inactivating TLR4 Signalling.” Phytomedicine 123: 155223. 10.1016/j.phymed.2023.155223. 38134862

